# Surface Binding Energy Landscapes Affect Phosphodiesterase Isoform-Specific Inhibitor Selectivity

**DOI:** 10.1016/j.csbj.2018.11.009

**Published:** 2018-12-28

**Authors:** Qing Liu, Andreas Herrmann, Qiang Huang

**Affiliations:** a State Key Laboratory of Genetic Engineering, MOE Engineering Research Center of Gene Technology, School of Life Sciences, Fudan University, Shanghai 200438, China; b Institute for Biology and IRI Lifesciences, Humboldt-Universität zu Berlin, Invalidenstrasse 42, Berlin 10115, Germany

## Abstract

As human phosphodiesterase (PDE) proteins are attractive drug targets, a large number of selective PDE inhibitors have been developed. However, since the catalytic sites of PDE isoforms are conserved in sequence and structure, it remains unclear how these inhibitors discriminate PDE isoforms in a selective manner. Here we perform long-time scale molecular dynamics (MD) simulations to investigate the spontaneous association processes of a highly selective PDE2A inhibitor (BAY60–7550) with the catalytic pockets of six PDE isoforms. We found that the free-energy landscapes of PDE:BAY60–7550 interactions on the PDE surfaces are very different between various PDE isoforms; and the free-energy landscape of PDE2A forms a favorable low-energy pathway that not only drives BAY60–7550 toward the target binding site, but also guides BAY60–7750 to adopt its native binding conformation known from crystal structure. Thus, this study reveals that the inhibitor interactions with the PDE surface residues play an important role in its high selectivity for PDE2A, and thereby provides new fundamental insights into the PDE isoform-specific inhibitor selectivity.

## Graphical Abstract

**Figure:**
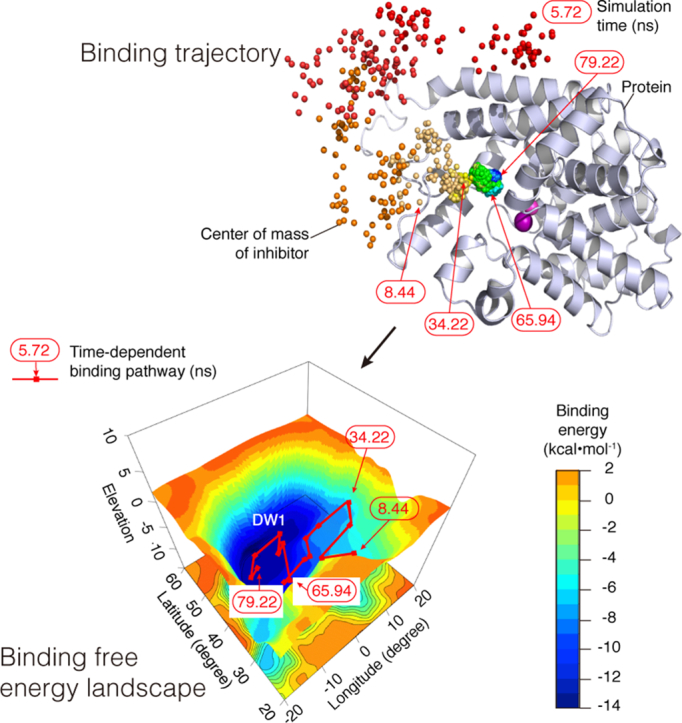

## 1 Introduction

Human phosphodiesterase (PDE) family consists of 11 subfamilies and expressed in a tissue-specific manner [1]. These PDE protein isoforms could hydrolyze the cyclic phosphate bonds of the secondary messengers cAMP and cGMP, and thereby terminate many signaling pathways in distinct human tissues [1]. Consequently, dysfunctions of the PDE isoforms are closely linked to different diseases [1], such as heart failure [2], schizophrenia [3], and cancer [4]. So the PDE proteins are important therapeutic targets. In the past two decades, many PDE inhibitors have been developed, including sildenafil (PDE5 inhibitor) [5], roflumilast (PDE4 inhibitor) [6], and milrinone (PDE3 inhibitor) [7], which were approved for the treatments of erectile dysfunction [8], chronic obstructive pulmonary disease (COPD) [6], and congestive heart failure [9], respectively .

**Fig. 1:**
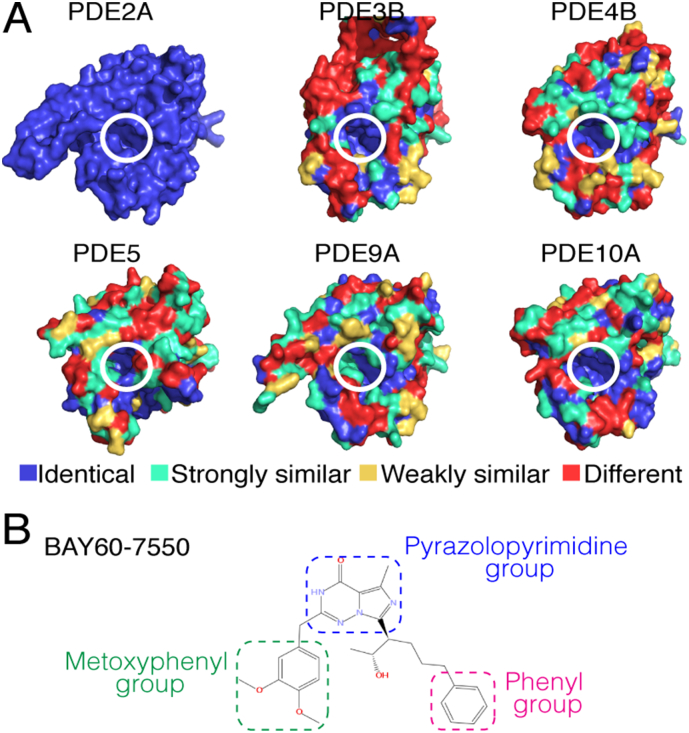
(A) Protein residue similarities of six PDE isoforms according to the multiple sequence alignment (MSA) in Fig. S1. Residues are classified into 4 groups: identical (blue), strongly similar (cyan), weakly similar (yellow), and different (red). Catalytic pockets of the six isoforms (white circles) mainly consist of the identical (blue) and strongly similar (cyan) residues. (B) BAY60–7550 molecule. (For interpretation of the references to colour in this figure legend, the reader is referred to the web version of this article.)

However, those PDE inhibitors could cause side effects during their clinical treatments. For example, the COPD drug roflumilast has the side effects of headache and nausea [6]. Such side effects may be attributed to that all the PDE isoforms share a very conserved structural fold and possess very similar residues at their active sites [10] (Fig. 1A), although their overall sequences are different (Fig. S1 in Supporting Information [SI] and Table 1). So the inhibitors cannot precisely distinguish their PDE targets from other isoforms. Because the binding selectivity of the PDE inhibitors is crucial for the development of safe drugs, it is necessary to fully understand the structural origins of the PDE inhibitor selectivity.

**Table 1:** PDE isoforms investigated in this study.

PDE isoforms	PDB ID	Sequence length (a.a.)	Identical residue	Strongly similar residue^a^	Weakly similar residue^a^	Sequence identity (%)	RMSDs to PDE2A (Å)^b^	IC_50_ [12] (nM)	ROSETTA docking score [11]
PDE2A	4HTX [11]	342	–	–	–	–	–	4.7 ± 1.0	−24.5
PDE3B	1SOJ [16]	420	93	72	42	49.3	2.13	> 4000	−21.3
PDE4B	4KP6 [17]	340	84	89	39	62.4	1.98	1830 ± 840	−18.6
PDE5	2H42 [18]	326	111	87	33	70.9	1.97	704 ± 148	−20.1
PDE9A	2HD1 [19]	326	89	88	44	67.8	1.92	> 4000	−19.5
PDE10A	2OUP [20]	331	114	76	28	65.9	1.87	940 ± 400	−21.5

a Residues are regarded as strongly or weakly similar if their scores in Gonnet PAM 250 matrix are greater or smaller than 0.5, respectively.

b RMSDs of alpha-C atoms of the superimposed PDEs with respect to PDE2A.

Recently, we showed that a sub-pocket in the PDE active sites may play an important role in the binding selectivity of a PDE2A inhibitor, BAY60–7550 (Fig. 1B) [11]. By solving its co-crystal structure with PDE2A, we revealed that BAY60–7550 binds to the PDE2A active site using a binding-induced, hydrophobic sub-pocket (i.e., H-pocket). Molecular docking showed that the H-pocket contributes significantly to the binding affinity, and thereby improve the BAY60–7550 selectivity for PDE2A. However, the docking also indicated that BAY60–7550 adopts similar poses and might induce H-pocket in other PDE isoforms. Since the H-pocket residues of these PDE isoforms are different from those of PDE2A [11], this might lead to slightly lower binding affinities in these PDE isoforms (see ROSETTA docking scores in Table 1). In fact, BAY60–7550 shows >100-fold selectivity for PDE2 compared to PDE5 and the other 4 PDEs tested (see IC_50_ in Table 1) [12]. So it is difficult to use the docking affinities alone to explain the experimental data in which BAY60–7550 displays higher selectivity for PDE2A than other PDE isoforms [12] (see also Table 1). Besides the H-pocket, it seems that other unknown factors also contribute to the high selectivity.

Meanwhile, some studies have demonstrated that the binding kinetic processes do also impact the drug efficacy [13,14]. This implies that all the protein residues involving in the binding and unbinding processes may play roles in the selectivity. Because the residues in the PDE catalytic pockets are highly conserved [10], it is natural to hypothesize that the high selectivity of BAY60–7550 for PDE2A might derive from those different residues those nonconserved residues outside of the PDE catalytic sites.

Inspired by the study of a cancer inhibitor (Dasatinib) [15], here we use long, unbiased molecular dynamics (MD) simulations to test our above hypothesis. To understand how the association processes affect the binding selectivity for various PDE isoforms, we investigate not only the binding process of BAY60–7550 to PDE2A, but also those to other 5 PDE isoforms in Table 1. In the simulations, no biased forces were applied to BAY60–7550 so that its movements from random surface positions toward the target binding pockets were completely driven by the interplay of the inhibitor, the PDE residues, and the solvent. The simulations successfully captured the association processes in which the inhibitor diffuses freely, and then spontaneously enters the catalytic pockets. Interestingly, we found that the binding energy landscapes that govern the association processes are very different for various PDE isoforms. These energy landscapes could guide BAY60–7550 to the target pockets by forming low-energy binding pathways. So this study reveals that the protein surface residues play an important role in the binding selectivity of BAY60–7550, and thus provides useful guidance on the rational design of the PDE selective inhibitors.

## 2 Materials and Methods

### 2.1 Set-Up of MD Simulation Systems

For the 6 isoforms in Table 1, we investigated the spontaneous association processes of BAY60–7550 with the PDE catalytic pockets from random surface positions.

The all-atom models of the 6 PDE catalytic domains were constructed from their crystal structures, respectively (PDB IDs: 4HTX, 1SOJ, 4KP6, 2H42, 2HD1, 2OUP) [11,[16], [17], [18], [19], [20]]. In these structures, the co-crystal ligands were removed, and the missing residues of the proteins were built by MODELLER (Ver. 9.11) [21] or I-TASSER (Ver. 5.1) [22]. Hydrogen atoms were added using PSFGEN (Ver. 1.6.3) [23]. In each of the six PDE:BAY60–7550 systems, four BAY60–7550 molecules (designated as inhibitor A, B, C, and D, respectively) were randomly placed outside the PDE protein according to the method described in SI Subsection S2 and Fig. S2. Then, this PDE-inhibitor system was immersed in a cubic water box with an edge length of ~100 Å and a salt concentration of 150 mM NaCl. The CHARMM36 force field [24] was used to describe the topology and interaction parameters of the PDE protein and the metal atoms (Mg^2+^ and Zn^2+^). The water molecules were described by the TIP3P model [25]. The topology and force-field parameters of BAY60–7550 were generated using SwissParam [26].

### 2.2 Unbiased MD Simulations

All simulations were performed with NAMD (Ver. 2.11) using the Langevin integrator [27,28]. Short-range van der Waals and electrostatic interactions were cut off at 10 Å. Periodic boundary conditions were employed; and the Particle Mesh Ewald (PME) method was used for long-range electrostatic interactions [29,30]. All bonds were constrained by the SHAKE algorithm [31]; so an integration time step of 2 fs was used. All simulated systems were first minimized for 1000 steps with the conjugate gradient descent algorithm. Then, the systems were equilibrated in the NVT ensemble for 20 ps at 320 K controlled by the Langevin thermostat [32]. The Langevin piston Nose-Hoover method was used to couple the system pressure to 1 atm [33,34]. Finally, unbiased production simulations were performed in the NPT ensemble for >80 ns. More details can be seen in SI Subsection S3.

### 2.3 Time-Dependent RMSD Calculation

We use root-mean-square deviation (RMSD) to evaluate the similarity between the PDE-bound conformations of BAY60–7550 in the simulations and the native pose in the catalytic pocket of PDE2A [11]. The time-dependent RMSD values were calculated for each of the four BAY60–7550 molecules in every simulation of a given PDE:BAY60–7550 system. For a given inhibitor (A, B, C, or D) in a specific simulation (1, 2, …, or 8), we designated the MD trajectory of the given inhibitor as ‘PDE isoform:Traj. simulation number - inhibitor ID’. For example, the trajectory of inhibitor A in simulation 1 of the PDE2A:BAY60–7550 system was named ‘PDE2A:Traj. 1-A'. For accurate comparison, before calculating RMSDs, all the MD snapshots of the PDE-inhibitor complex were superimposed on the crystal structure of PDE2A (PDB ID: 4HTX) using TM-align [35].

### 2.4 Binding Free Energy Calculation

In the MD simulations, a BAY60–7550 molecule was considered to be in the PDE-bound state if the distance from its center of mass (COM) to any surface residue is <5 Å, which is roughly equal to the inhibitor radius. To construct the binding free energy landscapes for all six PDE:BAY60–7550 systems, it is necessary to calculate the binding energies for vast numbers of MD snapshots, in which BAY60–7550 may be bound to any sits on the PDE surfaces. To reduce the computational costs, we employed a computationally inexpensive energy function, the AutoDock semiempirical free energy force field [36], to calculate the binding free energy for a given MD snapshot with the PDE-bound inhibitor. This function consists of molecular mechanics terms and empirical desolvation terms determined by linear regression analysis of complexes with known 3D-structures and known binding free energies; and, many studies have confirmed its ability to correctly predict the binding affinities of small molecules to proteins. So, we used this function to calculate the absolute free binding energy for moving BAY60–7550 from the common unbound state in bulk water (i.e., the initial reference state) to the final bound state specifically defined by the given MD snapshot (see more details in SI Subsection S5). Parameters of the protein, inhibitor and metal ions for the energy computations were calculated according to the standard procedures, with corresponding Python programs in AutoDockTools [37]. Meanwhile, we also used more rigorous, computational intensive method, free energy perturbation (FEP), to verify the free-energy values by the AutoDock function (see SI Subsection S11).

### 2.5 Construction of Binding Energy Landscapes

**Fig. 2:**
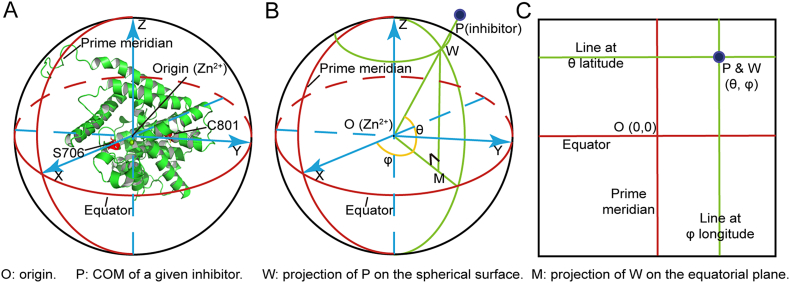
Coordinate systems for constructing the binding free energy landscapes. (A) The spherical coordinate system of PDE2A. (B) The transformation of the spatial coordinates into the geographic coordinates. (C) The unrolled 2D map.

To display the binding free energies for a given PDE:BAY60–7550 system, we treated the PDE2A catalytic domain as a sphere, and then used a geographical coordinate system to define the MD positions of the PDE-bound inhibitors. As shown in Fig. 2A, The Zn^2+^ ion in the PDE2A catalytic pocket was set as the origin; the X and Y axes are the lines connecting the origin to alpha-C atoms of S706 and C801 (PDE2A numbering), respectively; and the Z axis is determined by forming a right-hand system. To determine the positions of BAY60–7550 on the PDE surface, all the MD snapshots of the given PDE:BAY60–7550 complex were superimposed (see Fig. S3 in SI) onto the crystal structure of PDE2A in Fig. 2A, and then characterized using the coordinate system.

For the inhibitor at the given time of the simulations, we projected its COM at point P onto point W on the PDE surface (Fig. 2B). So the latitude of the inhibitor is the angle θ between the equatorial plane and the straight line joining the origin to W; the longitude is the angle φ between the prime meridian and the meridian passing through W. The signs of θ and φ indicate locations related to the equatorial plane and the prime meridian, respectively. For example, inhibitor with θ = 30 and φ = −60 is located at 30° N and 60° W in the geographic coordinates. Then, just like the World Map, the spherical surface is unrolled into a 2D flat map by converting the meridians and parallels to the vertical and horizontal lines, respectively (Fig. 2C). The 2D flat map was divided into 7200 unit grids of 3 × 3 square degrees. For each unit grid, exponential average binding energy was estimated. Finally, the discrete binding free energies on all unit grids were smoothed by thin-plate splines [38] and projected onto the 2D flat map in Fig. 2C FIELDS and PLOT3D packages in R (Ver. 3.3.3) [[39], [40], [41]] (see more details in SI Subsection S6).

### 2.6 Inhibitor Binding Probabilities to PDE Surfaces

To quantitatively characterize the binding probabilities of the inhibitor on different regions of the protein surface, we divided the 2D map (Fig. 2C) into 7200 unit grids of 3 × 3 square degrees, and counted the MD binding number of the inhibitor for each unit grid. These 7200 discrete binding numbers were directly used as the measure of the inhibitor binding probabilities to construct the binding probability surface on the 2D map,with the thin-plate spline method [38] (see more details in SI Subsection S7). All the binding probability surfaces were built using programs implemented in R (Ver. 3.3.3) [[39], [40], [41]].

### 2.7 Binding Pathway Mapping

For a successful MD trajectory of BAY60–7550 to the target binding pocket, we defined its binding pathway to the catalytic pocket as the time-dependent positions of the inhibitor in the PDE-bound state. To simplify the pathway display, only key positions in the binding process were connected by lines to represent the whole binding pathway in the given energy landscape.

## 3 Results and Discussion

### 3.1 Spontaneous Associations of BAY60–7550 with Target Binding Pockets

Totally, 48 MD simulations were performed for the 6 PDE isoforms (8 simulations per isoform). As mentioned, we used the RMSDs to evaluate the similarity between the PDE-bound conformations of BAY60–7550 in the MD simulations and the native pose [11]. Since the sizes of the PDE catalytic pockets are about 15.0 Å in diameter [42], we considered a simulation as a successful one that captures a binding event of the inhibitor from outside to the inside of the pocket, if anyone of the four inhibitors in the system associates with the pocket stably, and then fluctuates in the pocket with RMSDs <15.0 Å. Furthermore, we considered this PDE-bound inhibitor to be in an effective PDE-bound state to the catalytic pocket.

With the above criterion, for the 6 PDE:BAY60–7550 systems there are 4, 1, 2, 2, 3, and 3 successful simulations, respectively (Table 2); and the time-dependent RMSDs of the inhibitor in the corresponding trajectories are shown in Fig. S4 of SI. As shown, for each trajectory, when the inhibitor moves from outside random, distant positions to the inside of pockets with the simulation time, the RMSDs decrease rapidly from large values to smaller ones below 15.0 Å within ~60 ns. This clearly indicates that BAY60–7550 does possess a spontaneous tendency to associate with the catalytic pockets of the investigated PDEs.

**Table 2:** Summary of successful MD simulations, free energy landscapes, and binding probability (B.P.) of the PDE surfaces.

PDE isoforms	Successful Simulations (num.)	Minimal RMSDs (Å)	Binding energy of minimal- RMSD poses (kcal·mol^−1^)^a^	Landscape SDs (kcal·mol^−1^)^b^	Total DWs grids (num.)	DWs grids inside pocket (num., %)^c^	Total high B.P. grids (num.)^d^	High B.P. grids inside pocket (num., %)^e^	Snapshot inhibitors in effective binding (%)^f^
PDE2A	4	0.66	−12.47 ± 0.89	1.49	99	78, 78.79	11	7, 63.64	8.01
PDE3B	1	12.81	−8.55 ± 1.03	1.41	63	0, 0.00	29	0, 0.00	0.00
PDE4B	2	7.91	−6.71 ± 0.96	1.42	20	0, 0.00	19	0, 0.00	3.88
PDE5	2	0.79	−11.46 ± 0.84	1.28	83	49, 59.04	11	1, 9.09	7.79
PDE9A	3	7.45	−7.32 ± 0.81	1.32	54	6, 11.11	18	6, 33.33	7.22
PDE10A	3	6.37	−5.74 ± 0.73	1.22	5	0, 0.00	10	0, 0.00	3.53

a Exponential average binding energy of the snapshot inhibitors whose RMSDs are within 2 Å of the minimal RMSD (see also in SI Subsection S9).

b Maximum standard deviations of the average binding energy for each unit grid on the energy landscapes (see also SI Subsection S6).

c Number and percentage of the deep-well grids inside PDE pockets in total unit grids occupied by all deep wells.

d High binding probability (B.P.) grids are defined as those >360 num./grid, about 60% of the binding probability in the PDE2A catalytic pocket (~600 num./grid).

e Number and percentage of the high binding probability (B.P.) grids inside PDE pockets in all high binding probability (B.P.) unit grids.

f Percentage of the effective PDE-bound state inhibitors in all the snapshot inhibitors in the MD simulations.

**Fig. 3:**
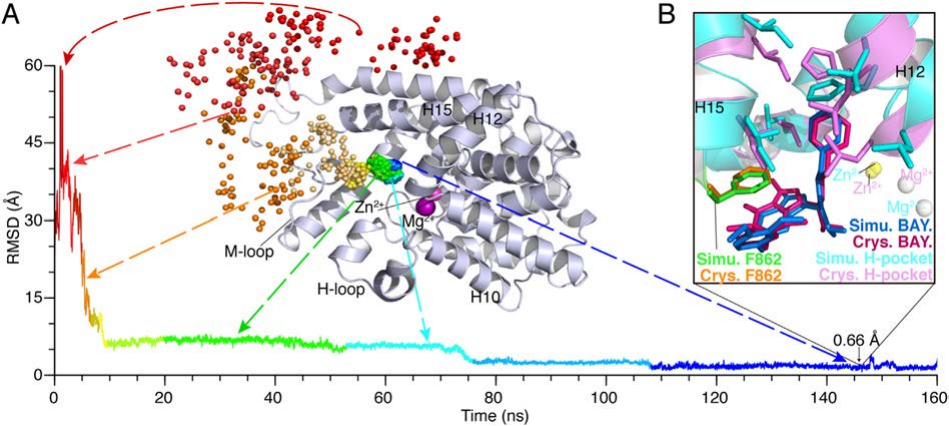
Spontaneous association of BAY60–7550 with the catalytic pocket in the MD trajectory PDE2A:Traj. 1-D. (A) The time-dependent RMSDs of BAY60–7550 with respect to the native pose from PDB 4HTX [11], and corresponding positions of the center of mass of BAY60–7550 (spheres in rainbow colors) on the protein (cartoon in gray). (B) The best-matched conformation of BAY60–7550 with the smallest RMSD of 0.66 Å at ~146 ns, where the H-pocket residues and F862 are represented as stick models. Corresponding movie showing the spontaneous association process is presented in Supporting Information (Supplementary Movie S1). Spontaneous association of BAY60–7550 with the catalytic pocket in the MD trajectory PDE2A:Traj. 1-D. (A) The time-dependent RMSDs of BAY60–7550 with respect to the native pose from PDB 4HTX [11], and corresponding positions of the center of mass of BAY60–7550 (spheres in rainbow colors) on the protein (cartoon in gray). (B) The best-matched conformation of BAY60–7550 with the smallest RMSD of 0.66 Å at ~146 ns, where the H-pocket residues and F862 are represented as stick models. Corresponding movie showing the spontaneous association process is presented in Supporting Information (Supplementary Movie S1).

Interestingly, we found that BAY60–7550 in the systems of PDE2A and PDE5 usually binds to the catalytic pockets within relatively short periods of ~25 ns, as compared to other 4 PDEs (Fig. S4 in SI). Also, in the simulations BAY60–7550 adopts binding poses that well match the native pose, with the minimal RMSDs of 0.66 and 0.79 Å, respectively (Fig. 3B); in contrast, the inhibitor in other 4 PDE systems achieves different binding poses with the minimal RMSDs of 6–8 Å (Table 2). So, in the simulation timescale, only the association processes in the PDE2A and PDE5 systems could lead BAY60–7550 to the native binding pose [11]. As we will further discuss in the subsections, this might suggest that BAY60–7550 is a good inhibitor for PDE2A and PDE5. Indeed, the IC_50_ data [12] (Table 1) have shown that the binding selectivity for PDE2A and PDE5 are better than those for other PDEs.

As expected, the best conformation of BAY60–7550 that matches the native pose was found in the simulations of PDE2A (i.e., PDE2A: Traj. 1-D in Fig. 3A); the final stable conformation of BAY60–7550 in this trajectory agrees very well with the native pose [11], with an RMSD of ~0.66 Å (Fig. 3B). Similar to the crystal structure, the major interactions stabilizing the bound conformation are: the π-π stack between phenylalanine of F862 and pyrazolo-pyrimidine group of BAY60–7550, and the H-pocket induced by the phenyl group of the inhibitor (Fig. 3B). As seen in Fig. 3, BAY60–7550 initially moves from a random position far away from the catalytic pocket to the M-loop near the pocket, then spontaneously enters the pocket along the M-loop, and eventually stays in the pocket in the native pose. As showed in Fig. S5, the time-dependent binding energy and RMSDs of this trajectory decrease in a similar manner and both converge finally. Moreover, the descents of RMSDs are almost be accompanied by the energy barriers. Three major energy barriers are labeled in Fig. S5. So, our MD simulations have captured the association dynamics of BAY60–7550 with its target binding site, and provide a solid basis for the following analyses.

**Fig. 4:**
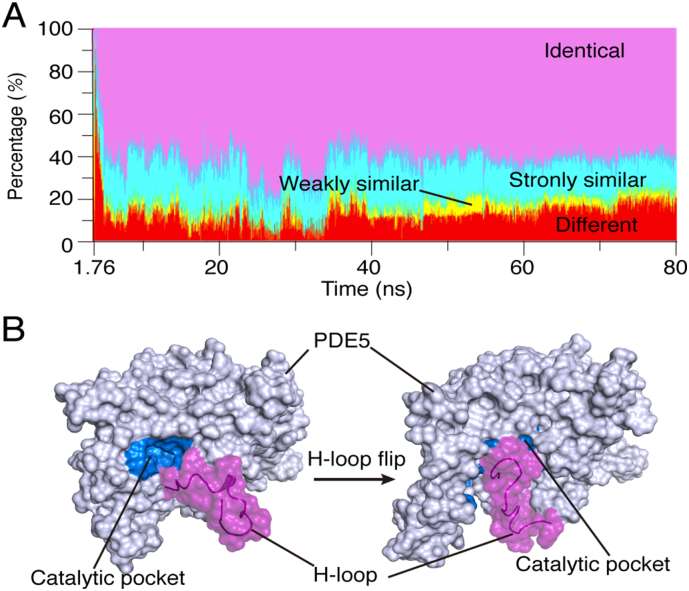
(A) Time-dependent similarity in inhibitor-bound residues of PDE2A and PDE5 in the MD trajectory PDE5:Traj. 8-B. The residue classification is the same as that in Fig. 1A. (B) The H-loop flip of PDE5 that blocks the catalytic pocket.

Another interesting trajectory is PDE5:Traj. 8-D, in which BAY60–7550 also matches the native pose well, with an RMSD of 0.79 Å (Fig. S6 in SI). In the simulation, the H-pocket is also induced by BAY60–7550, and the π-π stack is formed by F820 and the inhibitor, too (Fig. S6B in SI). Moreover, this MD trajectory is similar to that in PDE2A (Fig. 3A). Both time-dependent binding energy and RMSDs of this trajectory converge in a similar manner; and, several energy barriers accompany the descents of RMSDs (Fig. S7).We found that the residues involving in the association processes are quite conserved. As shown in Fig. 4A, about 80% of the PDE5 residues in the binding process are similar to those in PDE2A. It appears that the high similarity in the pathway residues is an important factor to lead the inhibitor to the catalytic pockets with the same conformation, namely, the native pose [11].

However, compared to PDE2A, the H-loop of PDE5 outside the pocket may reduce the probability of the inhibitor to correctly bind to the catalytic pocket. Unlike that in PDE2A, this loop is rather flexible to flip toward the entrance of the catalytic pocket during the simulations (Fig. 4B). Such an H-loop flipping occurred in 6 of the 8 PDE5 simulations, except for two successful trajectories in Fig. S4 of SI. So, if BAY60–7550 reaches the entrance after the H-loop flipping, it would be kept outside of the catalytic pocket during the rest time of the simulations. Consistent with the IC_50_ data [12], this also implies that BAY60–7550 is more selective for PDE2A than for PDE5. Indeed, the total number of successful trajectories in the PDE5 simulations is less than that of the PDE2A simulations (4 in Fig. S4 of SI).

In the simulations of other PDEs, the final binding poses of BAY60–7550 in the catalytic pockets are different from the native pose, with large RMSDs (> 6 Å) (Table 2 and Fig. S4 in SI). Also, these simulated poses fail to induce the H-pocket and to form π-π stacks with corresponding phenylalanines. In the absence of such two interactions, the minimal-RMSD poses in these 4 PDE isoforms are less stable than those in PDE2A and PDE5 (see the binding energy of minimal-RMSD poses in Table 2). This suggests that the binding selectivity values of BAY60–7550 for these PDEs are lower than those for PDE2A and PDE5, in agreement with the IC_50_ data in Table 1. Meanwhile, the differences between the simulated poses and the ROSETTA docking results indicate that the whole binding processes involve in the PDE2A-specific selectivity of BAY60–7550. The binding processes consist of complex protein-inhibitor interactions which can be monitored by the binding energy landscapes.

### 3.2 Distributions of Binding Energy Wells on PDE Surfaces

**Fig. 5:**
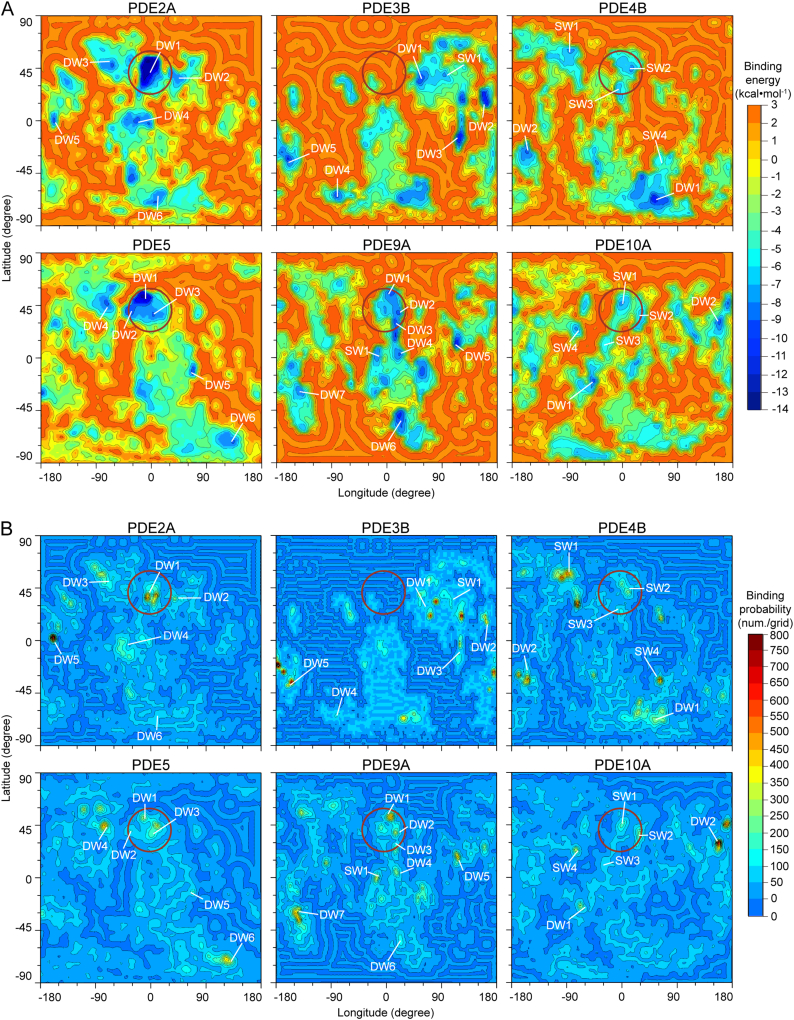
(A) The binding energy landscape of the 6 PDE:BAY60–7550 systems. (B) Corresponding binding probabilities. The catalytic pockets are indicated by red circles. Some deep energy wells (DWs, ΔG < −8.2 kcal·mol^−1^) and shallow energy wells (SWs, −8.2 < ΔG < −4.1 kcal·mol^−1^) on the maps are marked. (For interpretation of the references to colour in this figure legend, the reader is referred to the web version of this article.)

Energy landscape theory considers that the movement of an inhibitor on a protein are governed by the free energy profile of the inhibitor-protein interactions [43]. So the spontaneous association process of BAY60–7550 with its target binding pocket is ultimately determined by the so-called binding energy landscape of the inhibitor on the protein surface. Consistent with the RMSD results, the binding energy landscapes of the 6 PDE:BAY60–7550 systems are different (Fig. 5A). Again, this is attributed to their difference in the surface residues. As illustrated in Fig. 5A, all 6 energy landscapes are very rugged: almost half of the surface regions are energy ridges and hills (i.e., ΔG ≥ 0.0 kcal·mol^−1^), and other regions are energy basins and valleys (i.e., ΔG < 0.0 kcal·mol^−1^). For such regions, we defined the local energy minima < −4.1 kcal·mol^−1^ as energy wells, which corresponds to a *K*_d_ value <1000 μM. Similarly, the remaining regions from −4.1 to 0.0 kcal·mol^−1^ are regarded as energy barriers between the energy wells.

Because of the lower binding energies, the energy wells are hot spots that capture the inhibitor when it moves along the protein surface [44]. To better characterize the energy wells, we further divided them into two categories: deep well (DW) with a binding energy < −8.2 kcal·mol^−1^, which corresponds to a *K*_d_ value <1000 nM, and shallow well (SW) with a binding energy in the range from −8.2 to −4.1 kcal·mol^−1^. Generally, the depth of an energy well correlates strongly with the probability of the inhibitor trapped in this well. To characterize such inhibitor binding probability on the surface, we described it as the MD binding number to surface grids of 3 × 3 square degrees, in the unit of num./grid (see details in Subsection 2.6 and S7 in SI). Consistent with this, the inhibitor binding probabilities in the energy wells are generally higher than other areas (Fig. 5B).

We found that, in all 6 energy landscapes, the deepest and largest energy well is located at the catalytic pocket of PDE2A, i.e., DW1 with ΔG < −13.0 kcal·mol^−1^ (Fig. 5A). So the PDE-bound inhibitor in the PDE2A catalytic pocket may be considered as the most stable one in thermodynamics. Also, the DW1 area of PDE2A is the largest one among the deep wells inside the PDE catalytic pockets (78 grids, Table 2), and could increase the probability of the inhibitor to move into the catalytic pocket (Fig. 5B). Indeed, the binding probabilities in the catalytic pocket are the highest among all 6 PDE catalytic pockets (> 550 num./grid) (Fig. 5B). These indicate that the PDE2A catalytic pocket possesses the greatest capability to associate with BAY60–7550. Meanwhile, the number of deep energy wells outside the catalytic pocket is the least, since ~79% of grid occupied by the deep wells are located inside the PDE2A catalytic pocket (Table 2). Clearly, this is beneficial for the inhibitor to move quickly into the catalytic pocket, because the inhibitor is not easily trapped in the regions outside the catalytic pocket. This leads to that 7 of the 11 high B.P. grids are inside the PDE2A catalytic pocket (Table 2, Fig. 5B). Consequently, the inhibitor in 8% of the MD snapshots of the PDE2A:BAY60–7550 system is in the effective binding states to the catalytic pocket, the highest percentage in all 6 PDEs (Table 2).

In the vicinity of the PDE5 catalytic pocket, there are 3 energy wells: DW1, DW2, and DW3 (Fig. 5A). Compared to DW1 of PDE2A, they are shallower and smaller (49 deep-well grids inside the PDE5 catalytic pocket, Table 2), especially DW2 and DW3 (ΔG > −9.0 kcal·mol^−1^). So the catalytic pocket of PDE5 possesses relatively weak ability to associate with BAY60–7550, as also indicated by the binding probability of ~300 num./grid (Fig. 5B). In addition, the PDE5 energy landscape has higher proportion of deep-well area outside (~59% deep-well grids inside the PDE5 catalytic pocket, Table 2), leading to more high binding probability area outside the catalytic pocket (only ~9% high B.P. grids inside the PDE5 catalytic pocket, Table 2 and Fig. 5B). Together, the catalytic pocket of PDE2A appears to be a better binding site for BAY60–7550 than that of PDE5. In other words, PDE2A is more favorable for the binding of BAY60–7550 than PDE5, in agreement with the experimental data [12].

In the catalytic pockets of other 4 isoforms, the energy wells are shallower than those of PDE2A and PDE5, especially the catalytic pockets of PDE4B and PDE10A (ΔG > −8.0 kcal·mol^−1^, Fig. 5A). Meanwhile, these deep-well areas much smaller (0–6 the deep-well grids inside the catalytic pockets, Table 2 and Fig. 5A) than PDE2A and PDE5. Correspondingly, the binding probabilities in most these energy wells are smaller, too, especially those of PDE4B and PDE10A (< 250 num./grid, Fig. 5B). In addition, the proportion of deep-well area outside their catalytic pockets are much higher than those inside (only 0–16% of deep-well grids inside the catalytic pocket, Table 2 and Fig. 5A). As a result, landscape areas outside their catalytic pockets might have much stronger ability to trap inhibitors than those inside the areas. For example, there is no high B.P. grid inside the PDE4B catalytic pocket, but 19 high binding probability grids outside the pocket (Table 2 and Fig. 5B); and one of them has a binding probability about three times greater (> 550 num./grid) than that in the catalytic pocket (~200 num./grid). Thus, in the MD simulations of the PDE4B:BAY60–7550 system, the inhibitor population in the effective PDE-bound state is the very low (3.9% in Table 2).

The above analyses clearly show that the distinct binding energy landscapes dominate the distributions of binding energy wells on the PDE surfaces, and eventually the inhibitor binding probabilities on the surfaces. As a result, the MD populations of the inhibitors in the effective binding state are different in the catalytic pockets for the 6 PDEs. In principle, this difference is correlated with the residence times of the inhibitor in the catalytic pockets. Since the residence time is positively related to the selectivity of the inhibitors [45,46], the binding selectivity of BAY60–7550 for the 6 PDEs might be ranked as: PDE2A > PDE5 > other PDE isoforms. Again, this order agrees with the IC_50_ data (see Table 1).

### 3.3 Low-Energy Binding Pathways to Target Sites

**Fig. 6:**
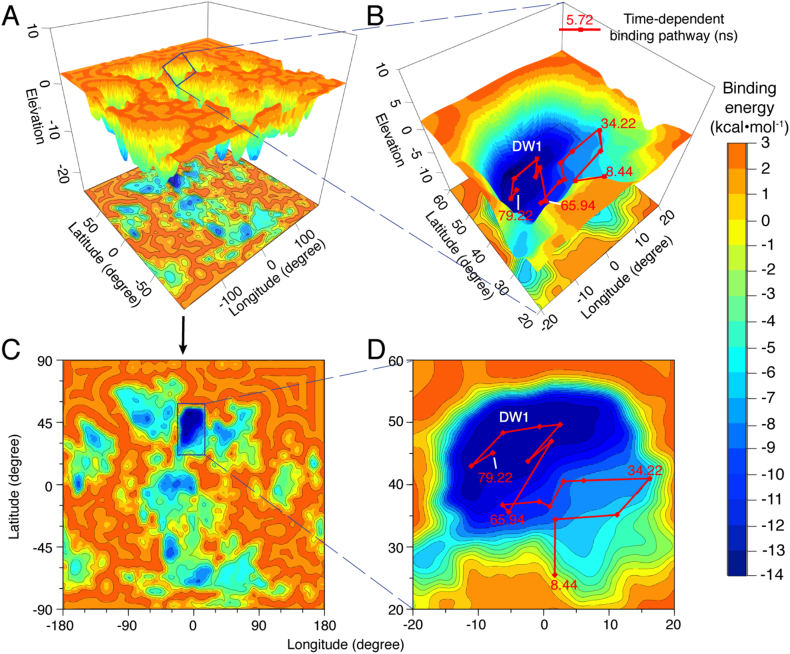
The binding pathway in PDE2A:Traj. 1-D. (A) The global 3D energy landscape of PDE2A. (B) The binding pathway in the 3D energy landscape. (C) The global energy landscape in the 2D map. (D) The binding pathway in the 2D map. The binding pathway (red lines) and time (red numbers) are shown on the energy landscape. The magnitudes of binding-energies are displayed in rainbow colors; and the deep energy well DW1 are labeled.

Since the energy wells are hot spots to associate with BAY60–7550, its association processes can be regarded as continuous movements from one energy well to another. To provide mechanistic insights, we map such binding pathways in the 6 PDE energy landscapes, as shown in Fig. 6 and S11-S24 in SI.

In PDE2A:Traj. 1-D, the binding pathway of BAY60–7550 starts from the DW1 boundary at about 8.44 ns, and eventually enters into DW1 (Fig. 6B and D). Because no high barrier exists in this process, BAY60–7550 moves quickly into the catalytic pocket, without significant conformational change, i.e., it binds to the catalytic pocket with the native pose. Clearly, before the binding to DW1, the inhibitor needs to adjust its conformation to the native pose. In fact, BAY60–7550 has already adopted the native pose when binding to the M-loop, as indicated by the RMSD values (Fig. 3A).

In PDE5:Traj. 8-B, the pathway begins from the southeast corner of the catalytic pocket, and travels through DW3, and then reaches DW1 (Fig. S18 in SI). Before crossing over the barrier between DW1 and DW3, the inhibitor stays in SW1 for about 45 ns, with RMSD values <3.0 Å (see also Fig. S6 in SI). This implies that DW3 is an important region for the inhibitor to adjust its conformation before entering into DW1. However, DW3 is often occupied by the H-loop, which may hamper the BAY60–7550 entrance into the catalytic pocket (Fig. 4B). Thus, it seems that the H-loop interrupts the conformational adjustments of BAY60–7550 in SW1.

Compared to two above pathways of PDE2A and PDE5, other pathways are very different. Other three pathways of PDE2A are located in the basin containing DW2 in first 20 ns and extend toward DW1 (Fig. S11~S13 in SI). The energy basin is located at the interspace between the helices H15 and H12, where significant conformational changes occur, as indicated by the RMSDs (Fig. S4 in SI). To reach DW1, these pathways climb over the energy barrier of about 0.0 kcal·mol^−1^ between DW1 and DW2 through the saddle, SD1 or SD2. Likely, the energy basin acts as a metastable area for the inhibitor to adjust its conformation for the binding to the catalytic pocket. Consequently, BAY60–7550 usually passes through DW2 instead of staying in this energy well, leading to smaller binding probabilities (~200 num./grid) in DW2 (Fig. 5B).

In the energy landscapes of PDE4B, PDE5, and PDE9A, their catalytic pockets contain 2 or more energy wells, and all of them are the destinations of the corresponding pathways. In that of PDE5, the pathway 3-A climbs over the energy barrier between DW1 and DW2, and reaches DW2 (Fig. S17 in SI). Two pathways in the PDE4B energy landscape start from 2 distinct sites and arrive at SW2 and SW3, respectively (Fig. S15~16 in SI). Even more, three pathways on the PDE9A energy landscape leads to 3 different destinations. The pathways 4-A and 6-D reach DW1 and DW2, respectively. But the pathway 2-A wanders between DW1 and DW2 (Fig. S19~21 in SI). Like the pathways 2-B of PDE2A, the pathway 6-D of PDE9A also passes through a metastable area including the energy wells SW1 and DW2~4 (Fig. S21 in SI).

The pathways on the energy landscapes of PDE10A wander in the catalytic pockets (S22~24 in SI). For example, the pathway 3-B on the PDE10A energy landscape wanders in the region of about 1800 square degrees (Fig. S22 in SI). This is attributed to the relatively shallow energy wells (−5.0 < ΔG < −4.0 kcal·mol^−1^) and the low energy barriers in the catalytic pockets. As a result, the trapped inhibitor can easily escape from these wells and further climb over the barriers. The singular pathway on the PDE3B surface even stops outside the catalytic pocket (Fig. S14 in SI). Although its time-dependent RMSDs converge under 15 Å (Fig. S4 in SI), the inhibitor actually bind to the interspace between helix H12 and H15 which is close to the PDE3B catalytic pocket. As a result, almost no inhibitor stably binds into the pocket (~0 effective binding snapshots in Table 2).

As described, the low-energy binding pathways reveal how the binding energy landscapes guide the spontaneous associations of the inhibitor with its target binding pockets. In these pathways, the inhibitor usually adjusts its conformations in the energy wells outside the catalytic pockets. Thus, in the final phases of the binding processes only subtle conformational changes are required for binding to the targeted sites. Therefore, both conformation selection [43] and induce-fit [47] occur in the associations of BAY60–7550 with the catalytic pockets.

## 4 Conclusions

In this study, we have performed unbiased MD simulations to successfully capture the spontaneous associations of BAY60–7550 with the catalytic pockets of the 6 PDE isoforms. In the simulations of PDE2A and PDE5, the final stable conformations binding to the pockets agree completely with the native pose revealed by our previous experiments [11], and the induced H-pocket was also correctly reproduced. We found that the PDE surface residues determine the distributions of the binding energy wells on the protein surfaces; and these wells form the favorable low-energy binding pathways of BAY60–7550 toward its target binding sites. Interestingly, for all 6 PDE isoforms, the binding pathways on PDE2A are most favorable for the inhibitor binding, and thereby explain the high selectivity of BAY60–7550 for PDE2A.

In structure-based drug design, a major method to improve the drug selectivity is to optimize its binding affinity for the target binding site, e.g., the active site of an enzymes [48]. However, it is difficult to use this rational approach when developing inhibitors for targeting a specific isoform in a large protein family, e.g., human protein kinase family with ~510 isoforms [49]. Because the isoforms in such a large family are often encoded by homologous genes for carrying out similar functions, usually their active sites are very conserved in the amino-acid sequences [49]. So, an inhibitor could bind to the active sites of various isoforms with similar interactions. Often, to optimize such interactions is not enough for an inhibitor to discriminate different isoforms. Our previous study [11] also showed that the molecular docking affinities alone could not explain the different selectivity values of BAY65–7550 for various PDE isoforms (see also Table 1).

In fact, our MD simulations have revealed that, except in PDE2A and PDE5, the binding of BAY60–7550 did not induce the H-pocket in other 4 PDE isoforms, which was found to occur in the molecular docking. This strongly suggests that molecular docking alone may be difficult for the design of the selective PDE inhibitors. Meanwhile, the binding affinity is a thermodynamic equilibrium measurement of the extent of the inhibitor bound to its receptor. And the thermodynamic equilibrium may not be reached or maintained in an open in vivo system, because the inhibitor concentrations in vivo vary faster than the binding and unbinding processes [14]. Indeed, structure-based drug design on the basis of the binding affinity has been found to have certain false-positive rates [50]. For example, the study by Biggin and co-workers [51] showed that the selectivity predictions of broad-spectrum inhibitor bromosporine have only modest accuracy.

As demonstrated by our work, to overcome such limitations, one may investigate the binding processes of the inhibitor to its target site. These processes are the time-dependent behaviors of the PDE-inhibitor complexes, e.g., the transition states [52]. Transition states are crucial to understand the inhibitor selectivity and to rationally optimize the inhibitor binding kinetics. For example, our unbiased, atomistic MD simulations and pathway analyses indicate that the M-loop, alpha-helix H12 and H15 of PDE2A play an important role in guiding BAY60–7550 into the catalytic pocket. So, further investigations on these factors might be useful for developing better selective inhibitors of PDE2A. In combination with the binding energy landscape analysis, the unbiased, long-time scale MD simulation is a promising strategy for the rational design of selective inhibitors, e.g., to identify selective inhibitors for a given protein isoform from a group of candidate molecules.

In conclusion, this study reveals that the high selectivity of BAY60–7550 for PDE2A is likely determined by its interactions with the PDE surface residues outside the target binding sites. Thus, our study provides new fundamental insights into the PDE inhibitor selectivity, and suggests an MD-based strategy for the rational design of the selective PDE inhibitors.

The following are the supplementary data related to this article.

Supplementary Movie S1 Movie showing the association of BAY60-7550 with the PDE2A catalytic pocket Supplementary Movie S1

Supplementary material 1 Image 1

Topology and parameters of BAY60-7550 Image 2
