# Supplementary material for: Surface Binding Energy Landscapes Affect Phosphodiesterase Isoform-Specific Inhibitor Selectivity
Source: Comput Struct Biotechnol J. 2018 Dec 28;17:101–9. doi: 10.1016/j.csbj.2018.11.009 (PMC6349013; doi:10.1016/j.csbj.2018.11.009)
Supplement: Supplementary file 2 — Supplementary material 1 [file mmc2.docx]

Supporting Information

**Surface Binding Energy Landscapes Affect Phosphodiesterase Isoform-Specific Inhibitor Selectivity**

Qing Liu,^†^ Andreas Herrmann^‡^ , Qiang Huang^†^*

^†^ *State Key Laboratory of Genetic Engineering, Engineering Research Center of Gene Technology of Ministry of Education, School of Life Sciences, Fudan University, Shanghai, 200438, China*

^‡^*Institute for Biology and IRI Lifesciences, Humboldt-Universität zu Berlin, Invalidenstrasse 42, 10115 Berlin, Germany*

***Table of Contents***

| S1. Amino-acid sequence alignment of 6 PDE isoforms  S2. Starting structures of PDE:BAY60-7550 systems for MD simulations  S3. Parameters for MD simulations  S4. Superimposed PDE structures on PDE2A  S5. AutoDock semi-empirical free energy function  S6. Construction of binding free energy landscapes  S7. Construction of inhibitor binding probability maps  S8. Time-dependent RMSDs of BAY60-7550 in successful MD trajectories  S9. Time-dependent binding energy of PDE2A:traj 1-D  S10. Spontaneous association of BAY60-7550 with PDE5 catalytic pocket  S11. Binding free energy calculation by free energy perturbation (FEP)  S12. Summary of calculated binding energies  S13. Binding pathways of BAY60-7550 in successful MD trajectories  S14. Movie showing BAY60-7500 association with PDE2A catalytic pocket  References | 2  2  3  4  4  5  5  7  8  9  10  12  13  27  28 |
| --- | --- |

**Corresponding author**: huangqiang@fudan.edu.cn

S1. Amino-acid sequence alignment of 6 PDE isoforms

**
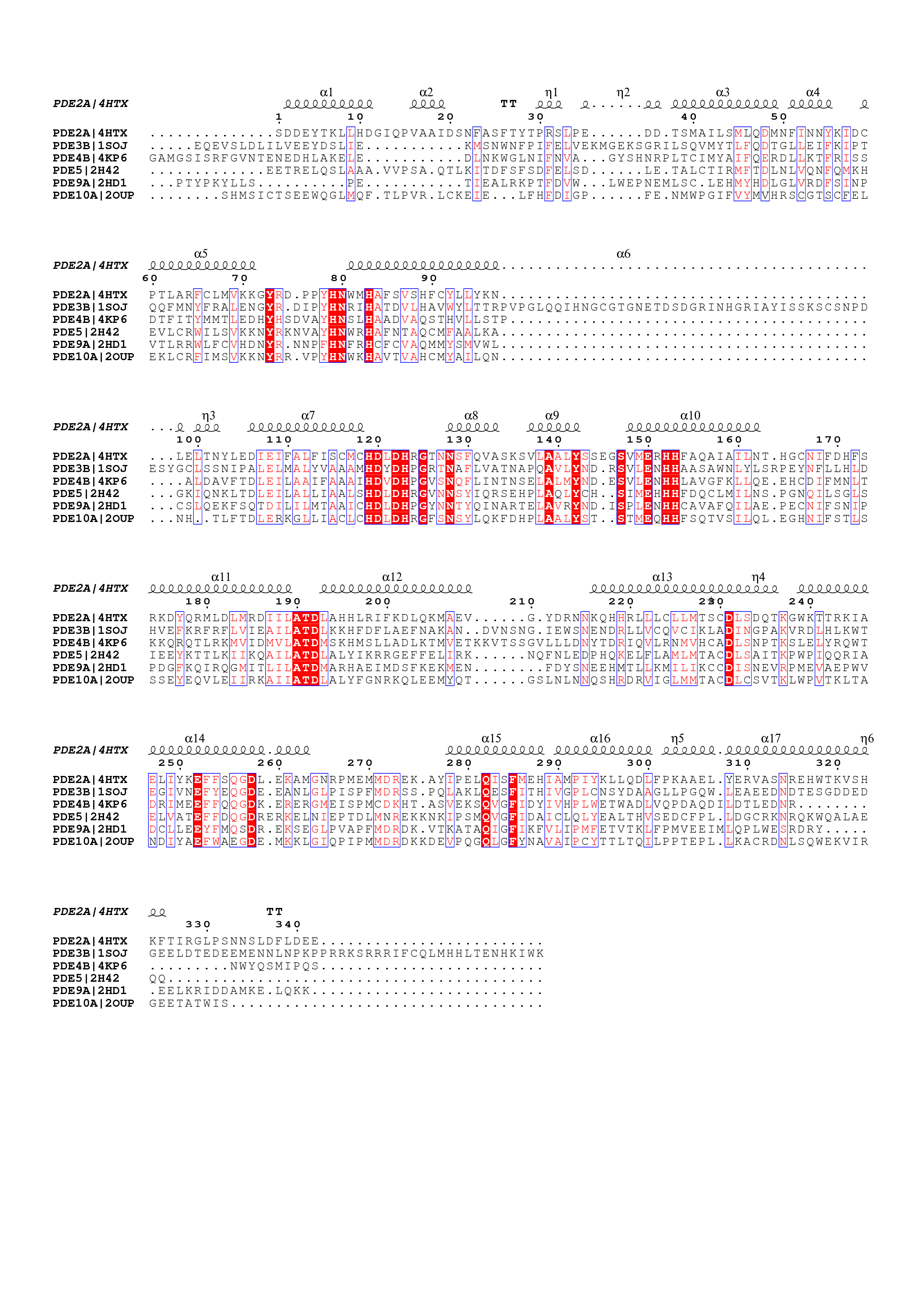
**

**Fig. S1.** Multiple sequence alignment (MSA) of 6 studied PDE isoforms using Espript (Ver. 3.0).[[1](#_ENREF_1)]

S2. Starting structures of PDE:BAY60-7550 systems for MD simulations

The MD starting structures for the 6 PDE:BAY60-7550 systems were built using the crystal structures of their catalytic domains (see corresponding PDB IDs in Table 1). As illustrated in Fig. S2, we set the center of mass (COM) of the catalytic domain of a given PDE (cyan) as the origin of the Cartesian coordinate system. Then, four BAY60-7550 molecules (designated as inhibitor A, B, C, and D, respectively) were randomly placed on the X and Y axes, ensuring that any molecule was at least 8 and 30 Å away from the PDE surface and the COM, respectively, by considering that the first primary energetic barrier might be 15 Å away from the catalytic sites.[[2](#_ENREF_2)] These two parameters ensure that the inhibitors need to climb over the energetic barrier before entering into the PDE catalytic pockets, and also give the MD systems proper sizes accessible to computational costs. The initial conformation of BAY60-7550 was set as the native pose in the crystal structure (PDB ID: 4HTX).[[3](#_ENREF_3)]

| 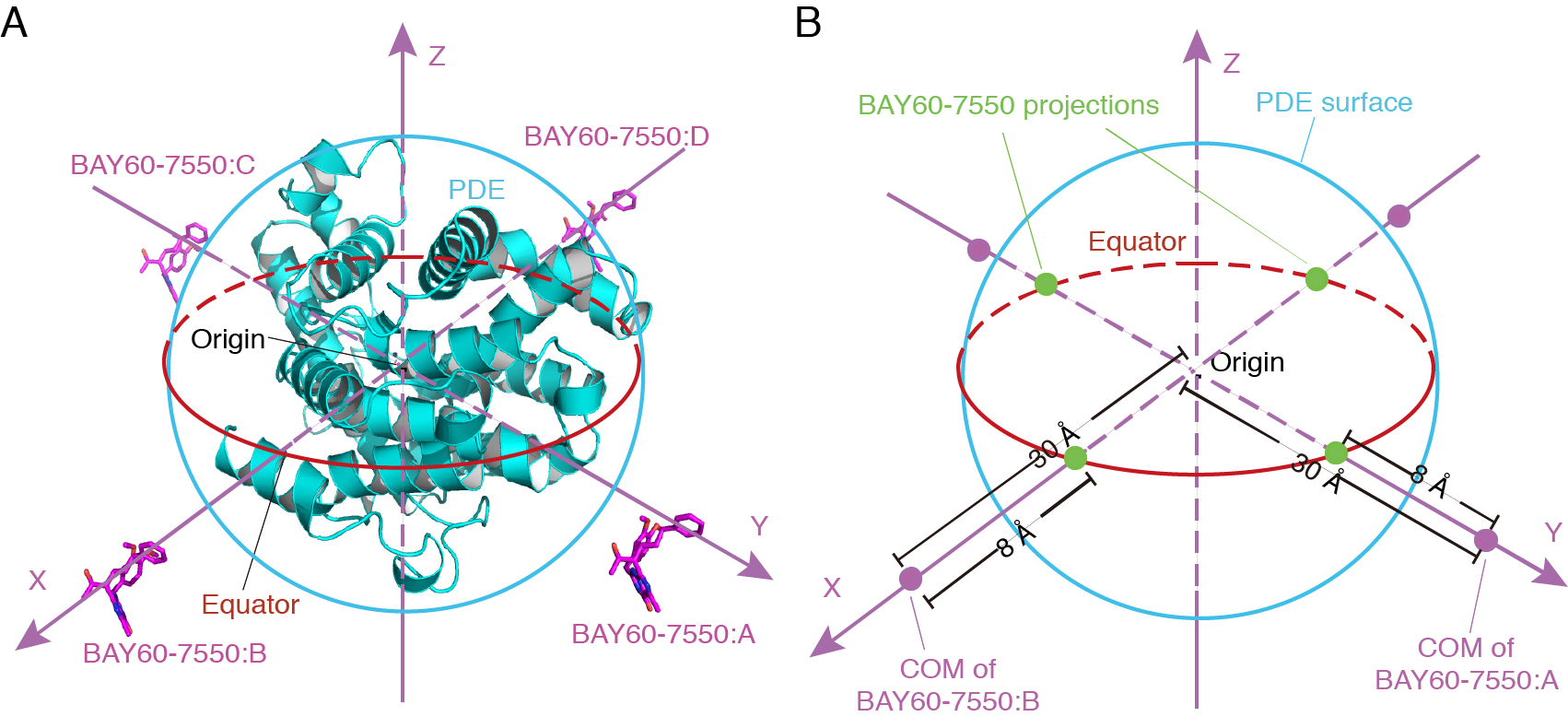 |
| --- |
| **Fig. S2.** MD starting structure of a given PDE:BAY60-7550 system. (A) Positions of the PDE catalytic domain (cartoon model in cyan) and four BAY60-7550 molecules (stick models in purple)[[4](#_ENREF_4)], with the COM of the catalytic domain as the coordinate origin. (B) The distances from BAY60-7550 to the PDE surface and the origin, respectively. |

S3. Parameters for MD simulations

| Table S1. Parameters for the MD simulations. | |  |  |
| --- | --- | --- | --- |
| Parameters | Values | Parameters | Values |
| Box sizes (x/y/z Å) | 104.5/101.3/74.7 | Langevin temperature (K) | 320 |
| PME tolerance[[5](#_ENREF_5)] | 1×10^-6^ | Langevin damping coefficient (ps^-1^)[[6](#_ENREF_6)] | 1 |
| System size (atom number) | ~7.4×10^4^ | Langevin dynamics applied to hydrogens | off |
| PME Ewald coefficient[[5](#_ENREF_5)] | 0.312341 | Langevin piston pressure (bar)[[6](#_ENREF_6)] | 1.01325 |
| PME interpolation order[[5](#_ENREF_5)] | 4 | Oscillation period (fs) | 100 |
| PME grid dimensions (x/y/z Å)[[5](#_ENREF_5)] | 100/100/75 | Decay time (fs) | 50 |
| Rigid bonds to hydrogen | all | Piston temperature (K) | 320 |
| Bond-length error tolerance | 1×10^-8^ | Pressure control | Group-based |
| SHAKE maximum iterations[[7](#_ENREF_7)] | 100 | Cell fluctuation | Iostropic |
| Use SETTLE for waters | on |  |  |

S4. Superimposed PDE structures on PDE2A

| 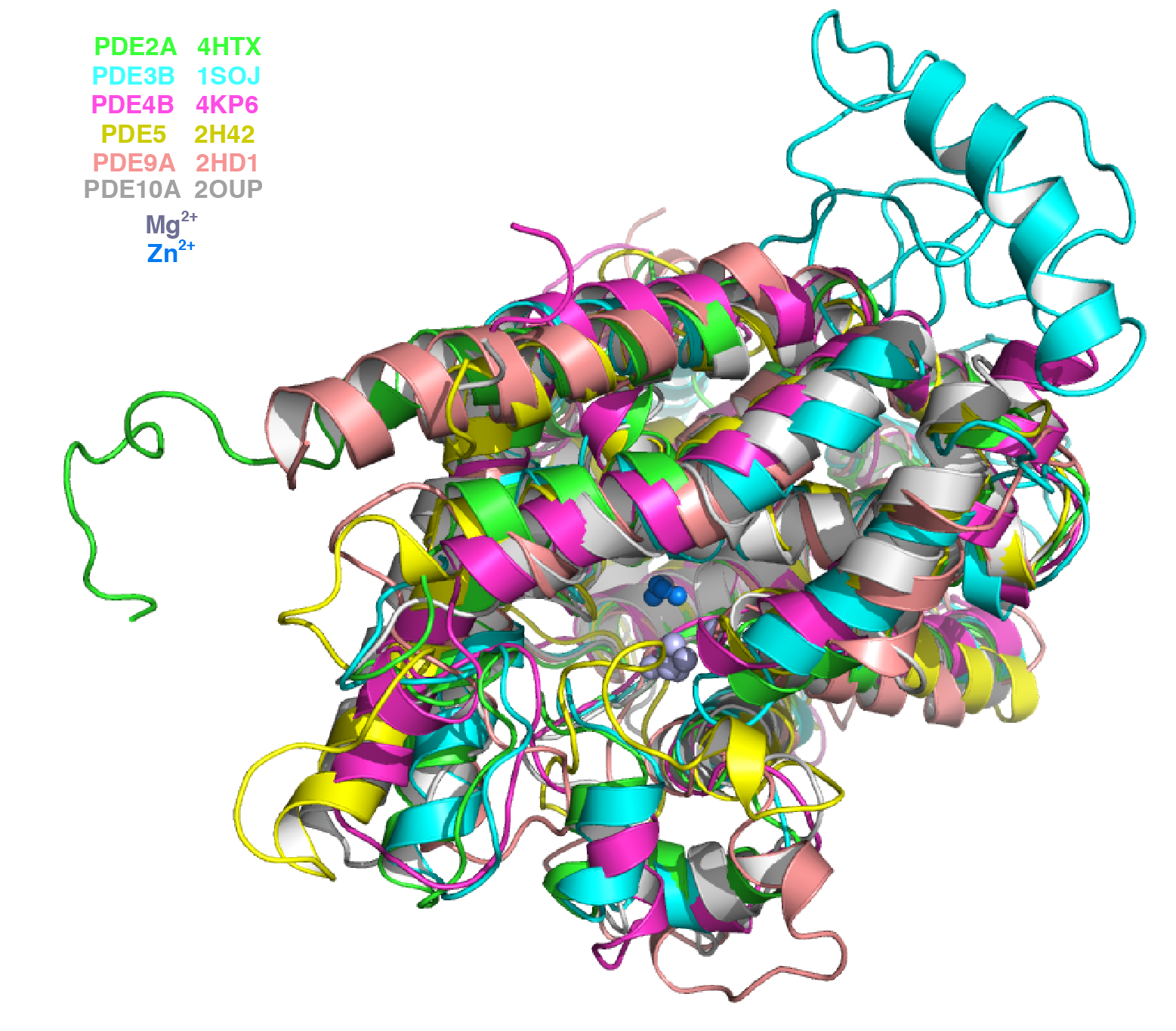 |
| --- |
| **Fig. S3.** The final simulated snapshots of 5 PDEs are superimposed on the crystal structure of PDE2A (PDB ID: 4HTX) using TM-align.[[8](#_ENREF_8)] |

S5. AutoDock semi-empirical free energy function

The binding free energy calculation by AutoDock is divided into two steps: (i) to evaluate the intramolecular energetics of the transition from the unbound state to the bound conformation for each of the molecules separately; and (ii) then to calculate the intermolecular energetics of bringing the two molecules together into the bound complex.[[9](#_ENREF_9)] In both steps, the energy is computed using a semi-empirical free energy force field that includes six pairwise terms ($V$) and a term for the conformational entropy lost upon binding ($\Delta S_{conf}$):[[9](#_ENREF_9), [10](#_ENREF_10)]

|  | ${\Delta G}_{bind}={(V}_{unbound}^{L-L}-V_{bound}^{L-L})+(V_{unbound}^{P-P}-V_{bound}^{P-P})+(V_{unbound}^{P-L}-V_{bound}^{P-L}+\Delta S_{conf}).$ | (1) |
| --- | --- | --- |

where *L* and *P* represent the ligand and protein, respectively. Each pairwise atomic terms in equation (1) include calculations for dispersion/repulsion, hydrogen bonding, electrostatics, and desolvation terms:[[9](#_ENREF_9), [10](#_ENREF_10)]

|  | $V=W_{vdw}\sum_{i,j} \left( \frac{A_{ij}}{r_{ij}^{12}}-\frac{B_{ij}}{r_{ij}^{6}} \right)+W_{hbond}\sum_{i,j} E\left( t \right)\left( \frac{C_{ij}}{r_{ij}^{12}}-\frac{D_{ij}}{r_{ij}^{10}} \right)+W_{electric}\sum_{i,j} \frac{q_{i}q_{j}}{\varepsilon\left( r_{ij} \right)r_{ij}}+W_{solv}\sum_{i,j} \left( S_{i}V_{j}+S_{j}V_{i} \right)e^{\left( {-r_{ij}^{2}}/{2\sigma^{2}} \right)}$. | (2) |
| --- | --- | --- |

In equation (2), most parameters are same as the ones in traditional molecular mechanics force fields. For example, the $A_{ij}$ and $B_{ij}$ in the first term come from the AMBER force field.[[11](#_ENREF_11)] Meanwhile, this semi-empirical force field uses an empirical method to estimate the desolvation free energy, e.g., the last term in equation (2).[[12](#_ENREF_12), [13](#_ENREF_13)] Finally, the weighting constants W in the equation (2) are optimized to calibrate the empirical free energies of experimentally characterized complexes. [[9](#_ENREF_9), [10](#_ENREF_10)]

S6. Construction of binding free energy landscapes

As described in Materials and Methods, for a given PDE:BAY60-7550 system all the binding free energies of the PDE-bound inhibitors in the MD snapshots were calculated with AutoDockTools using the semi-empirical energy functions of AutoDock 4.1.[[10](#_ENREF_10)] We used this discrete data set of energy values to construct the binding free energy landscape of PDE:BAY60-7550 interactions on the protein surface represented by the 2D flat map in Fig. 2C. This binding free energy landscape $f$ is described as a surface function:

|  | $\Delta G=f\left( \theta,\varphi\right)$, | (3) |
| --- | --- | --- |

where $\Delta G_{bind}$ is the binding free energy of the inhibitor to PDE, $\theta$ and $\varphi$ are the latitude and longitude of the inhibitor binding position on the PDE surface, respectively. So the obtained energy data set at *N* binding positions is described as: $S_{MD}=\left\{ {\Delta G}_{i}=f\left( \theta_{i},\varphi_{i} \right) | i=1,2,\ldots,N \right\}$.

Then, we divided the 2D flat map in Fig. 2C into 7,200 unit grids of 3×3 square degrees, and assigned a binding energy for each unit grid in this way: if in the data set *S_MD_* certain *m* energy values were found in the *j*th unit grid, the binding free energy for this unit grid was the exponential average of those *m* energy values; if no, a binding energy of 2 kcal∙ mol^-1^ was assigned for the unit grid. The exponential average binding energy of the *j*th unit grid is:

|  | $\left\langle\Delta G_{j} \right\rangle=\frac{\sum_{k=1}^{m} \Delta G_{j_{k}}exp\left( -\Delta G_{j_{k}}/k_{B}T \right)}{\sum_{k=1}^{m} exp\left( -\Delta G_{j_{k}}/k_{B}T \right)}$. | (4) |
| --- | --- | --- |

The energy standard deviations of the *j*th unit grid is:

|  | $\sigma_{\Delta G_{j}}=\sqrt{\left\langle\left( \Delta G_{j_{k}}-\left\langle\Delta G_{j} \right\rangle\right)^{2} \right\rangle}=\sqrt{\frac{\left( \sum_{k=1}^{m} \left( \Delta G_{j_{k}}-\left\langle\Delta G_{j} \right\rangle\right)^{2}exp\left( -\Delta G_{j_{k}}/k_{B}T \right) \right)}{\sum_{k=1}^{m} exp\left( -\Delta G_{j_{k}}/k_{B}T \right)}}$. | (5) |
| --- | --- | --- |

In equations (4) and (5), $k_{B} \mathrm{and} T$ are Boltzmann constant and temperature in Kelvin, respectively. So the gridded data set of the binding free energies covering the whole PDE surface was obtain as:$S_{grid}=\left\{ {\Delta G}_{i}=\overline{f}\left( \theta_{i},\varphi_{i} \right) | i=1, 2,\ldots, 7200 \right\}$, where $\overline{f}$ is the exponential average binding free energy landscape in the unit grids. Finally, we employed thin-plate splines[[14](#_ENREF_14)] to smooth $S_{grid}$ with the programs implemented in R[[15-17](#_ENREF_15)] to yield the smooth energy landscape $\hat{f}$ for the given PDE:BAY60-7550 system:

|  | $\Delta G_{i}=\hat{f}\left( \theta,\varphi\right)$ | (6) |
| --- | --- | --- |

Those smoothed values of $\Delta G_{bind}$ are represented as elevations in the 3D energy landscape and rainbow colors on the 2D flat map, respectively.

S7. Construction of inhibitor binding probability maps

As described in Results and Discussion, low-energy regions ($\Delta G_{bind}$< -4.1 kcal∙mol^-1^) in a binding energy landscape are hot spots to associate with the inhibitor. So the inhibitor binds to these regions with relatively high probabilities. To quantitatively characterize such inhibitor binding probabilities, we describe the inhibitor binding probabilities as a surface function on the 2D flat map:

|  | $P=g\left( \theta,\varphi\right)$ | (7) |
| --- | --- | --- |

where *P* is the binding probability of the inhibitor to PDE, $\theta$ and $\varphi$ are the latitude and longitude of the inhibitor binding position, respectively.

Similar to the treatments for the binding free energy landscapes, we also divided the 2D map in Fig. 2C into 7,200 unit grids of 3×3 square degrees, and then counted the number of the snapshot inhibitors binding to each unit grid in the MD simulations to obtain a gridded data set of 7,200 inhibitor binding numbers as: $S_{bind}=\left\{ P_{i}=g\left( \theta_{i},\varphi_{i} \right) | i=1, 2,\ldots, 7200 \right\}$. For the sake of simplicity, we directly used the inhibitor binding numbers as the measure of the binding probabilities on the PDE surface. Again, we employed thin-plate splines[[14](#_ENREF_14)] to smooth $S_{bind}$ with the programs implemented in R[[15-17](#_ENREF_15)] to yield the smooth inhibitor binding probability map $\hat{g}$ for the given PDE:BAY60-7550 system, as:

|  | $P=\hat{g}\left( \theta,\varphi\right)$ | (8) |
| --- | --- | --- |

Those smoothed values of *P* are represented as rainbow colors on the 2D flat map.

S8. Time-dependent RMSDs of BAY60-7550 in successful MD trajectories

| 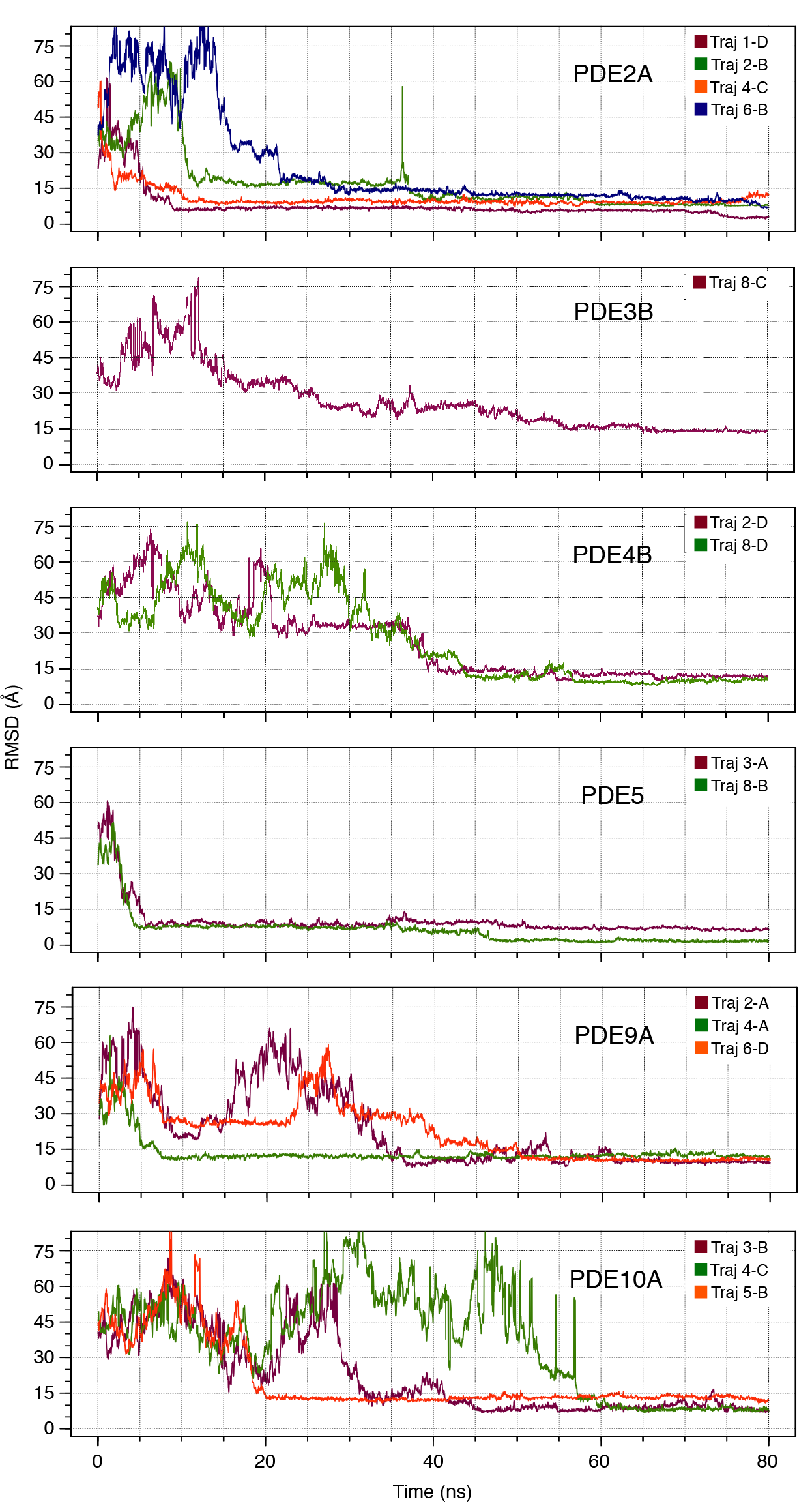 |
| --- |
| **Fig. S4.** Time-dependent RMSDs of BAY60-7550 in the successful MD trajectories in which BAY60-7550 binds to the target catalytic pockets with fluctuated RMSDs < 15 Å. |

S9. Time-dependent binding energy of PDE2A:traj 1-D

| 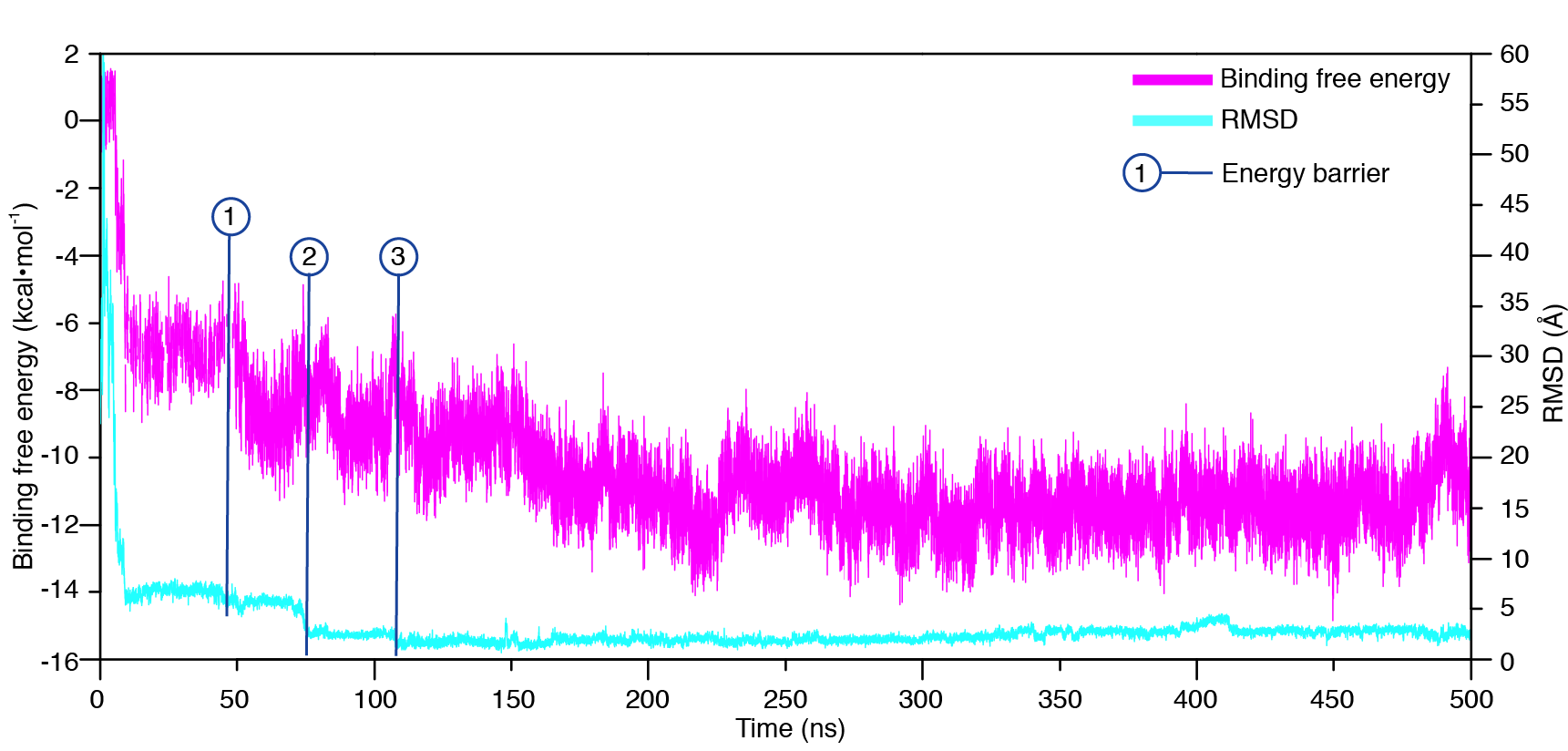 |
| --- |
| **Fig. S5.**  Time-dependent binding energy and RMSDs of the MD trajectory PDE2A:traj 1-D. Three major energy barriers are marked. |

Fig. S5 shows that the time-dependent binding energy and RMSDs of PDE2A:traj. 1-D decrease in a similar manner and both converge finally. Moreover, the descents of RMSDs are almost accompanied by the energy barriers. To evaluate the binding free energy of the stable binding pose in this trajectory, we calculated the exponential average binding energy by:

|  | $\Delta G_{bind}=\left\langle\Delta G_{stable} \right\rangle=\frac{\sum_{i=1}^{n} \Delta G_{i}exp\left( -\Delta G_{i}/k_{B}T \right)}{\sum_{i=1}^{n} exp\left( -\Delta G_{i}/k_{B}T \right)}$. | (9) |
| --- | --- | --- |

and the standard deviation was estimated by:

|  | $\sigma_{\Delta G_{bind}}=\sqrt{\left\langle\left( \Delta G_{i}-\left\langle\Delta G_{stable} \right\rangle\right)^{2} \right\rangle}=\sqrt{\frac{\left( \sum_{i=1}^{n} \left( \Delta G_{i}-\left\langle\Delta G_{stable} \right\rangle\right)^{2}exp\left( -\Delta G_{i}/k_{B}T \right) \right)}{\sum_{i=1}^{n} exp\left( -\Delta G_{i}/k_{B}T \right)}}$. | (10) |
| --- | --- | --- |

where $\Delta G_{i}$ is the binding free energy for the stable binding poses in PDE2A:traj. 1-D. These poses are the MD snapshots that have the RMSDs within 2 Å of the minimal RMSD, 0.66 Å. The $\Delta G_{bind}$ and $\sigma_{\Delta G_{bind}}$were also estimated for other 5 PDE systems.

S10. Spontaneous association of BAY60-7550 with PDE5 catalytic pocket

| 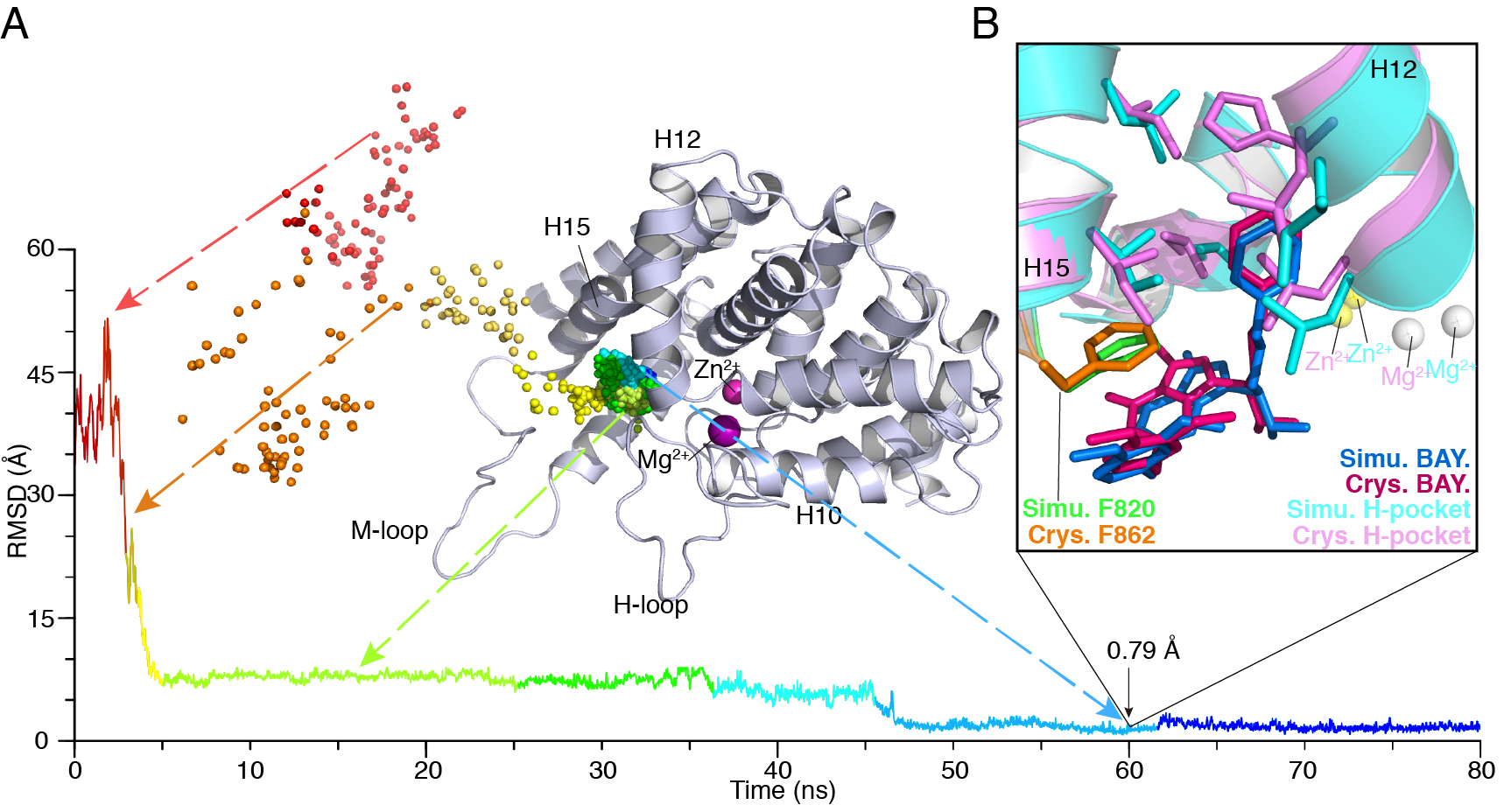 |
| --- |
| **Fig. S6.** Spontaneous association of BAY60-7550 with the catalytic pocket in the MD trajectory PDE5:Traj. 8-B. (A) The time-dependent RMSDs of BAY60-7550 with respect to the native pose in PDB 4HTX,[[3](#_ENREF_3)] and corresponding positions of its center of mass (COM) (spheres in rainbow colors) on the protein (cartoon in gray).[[4](#_ENREF_4)] (B) The best-matched pose of BAY60-7550 with the smallest RMSD of 0.79 Å at ~59 ns, where the H-pocket residues and F820 are represented as stick models. |

| 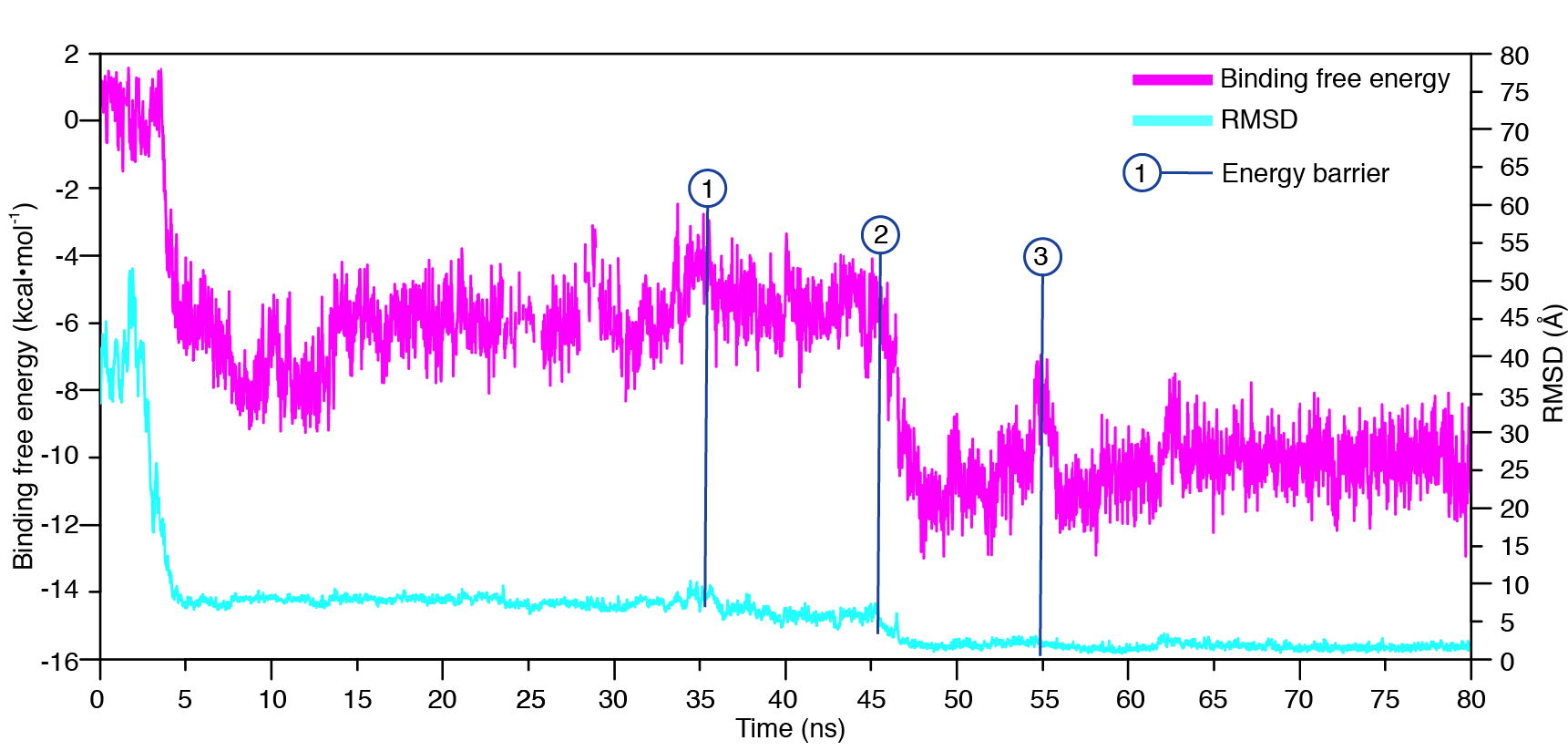 |
| --- |
| **Fig. S7.** Time-dependent binding energy and RMSDs of the MD trajectory PDE5:traj 8-B. Three major energy barriers are marked. |

S11. Binding free energy calculation by free energy perturbation (FEP)

To verify the reliability of the binding energies calculated with the AutoDock function, we performed FEP calculations[[18](#_ENREF_18)] to estimate the binding free energies of the minimal-RMSD poses in 5 PDE systems. For each PDE system, the minimal-RMSD BAY60-7550 in complex with PDE was chosen to be the start structure in the of FEP calculations. All MD simulations were carried out in GROMACS (Ver. 5.1.2)[[19](#_ENREF_19)]. The van der Waals interactions and the Coulombic interactions of the inhibitor were gradually restored using a linear alchemical pathway with Δλ = 0.05. To avoid the system collapse, the van der Waals interactions were gradually turned on while the Coulombic interactions were kept off, by setting the parameters in MDP files as:

vdw_lambdas = 0.00, 0.05, 0.10, ... ,1.00, 1.00, 1.00, ... ,1.00

coul_lambdas = 0.00, 0.00, 0.00, ... ,0.00, 0.05, 0.10, ... ,1.00

The λ value for the Coulombic interactions is always zero while the λ value for transforming the van der Waals interactions changes. Then, the van der Waals interactions are fully on (λ = 1.00) while the Coulombic interactions are gradually turned on. As a result, 41- window simulations for both the complex and ligand simulations were performed. For each window, 5,000 energy minimization steps were carried out using the steepest descent algorithm. The system was subsequently simulated for 100 ps in both the NVT ensemble and NPT ensemble with harmonic position restraints applied to the solute heavy atoms. Temperature was coupled using Langevin dynamics with 320 K as the reference temperature.[[20](#_ENREF_20), [21](#_ENREF_21)] Pressure was coupled using the Berendsen weak coupling algorithm with a target pressure of 1 atm.[[22](#_ENREF_22)] Then, a 5 ns unrestrained production run was performed for each window of the complex and ligand simulations (see more details in Table S2). The free energy changes of the complexation ΔG_complexation_ and the ligand solvation ΔG_solvation_ were calculated from the complex and ligand simulations, respectively. These were done by the GROMACS tool g_bar based on the Bennett Acceptance Ratio (BAR) method.[[23](#_ENREF_23)] Finally, the binding free energy was obtained as:

|  | $\Delta G_{bind}^{FEP}=\Delta G_{complexation}-\Delta G_{solvation}$. | (11) |
| --- | --- | --- |

| Table S2. | |  |  |
| --- | --- | --- | --- |
| Parameters for the FEP calculations. | |  |  |
| Parameters | Values | Parameters | Values |
| Time step (fs) | 2 | Pressure coupling method | Parrinello−Rahman[[24](#_ENREF_24)] |
| Integrator | Langevin dynamics[[22](#_ENREF_22)] | Reference pressure (atm) | 1 |
| Neighbor cutoff scheme | Verlet[[25](#_ENREF_25)] | Harmonic force constant (kJ·mol^-1^·nm^-2^) | 1000 |
| Neighbor search method | Grid | Rigid bonds to hydrogen | all |
| Neighbor list cut-off (Å) | 12 | Bonds constraint algorithms | LINCS |
| PME interpolation order[[5](#_ENREF_5)] | 6 | LINCS order[[26](#_ENREF_26)] | 12 |
| PME grid spacing (Å)[[5](#_ENREF_5)] | 1.2 | van der Waals interaction type | Cut-off |
| PME tolerance[[5](#_ENREF_5)] | 1×10^-6^ | Periodic boundary conditions | All directions |
| Coulomb cut-off (Å) | 12 | Soft-core alpha parameter[[27](#_ENREF_27)] | 0.5 |
| Distance for LJ cut-off (Å) | 12 | Power of radial term in soft-core function[[27](#_ENREF_27)] | 1 |
| Temperature coupling | Langevin dynamics[[22](#_ENREF_22)] | Soft-core sigma (nm)[[27](#_ENREF_27)] | 0.3 |
| Reference temperature (K) | 320 | Molecule for coupling free energy | BAY60-7550 |

| 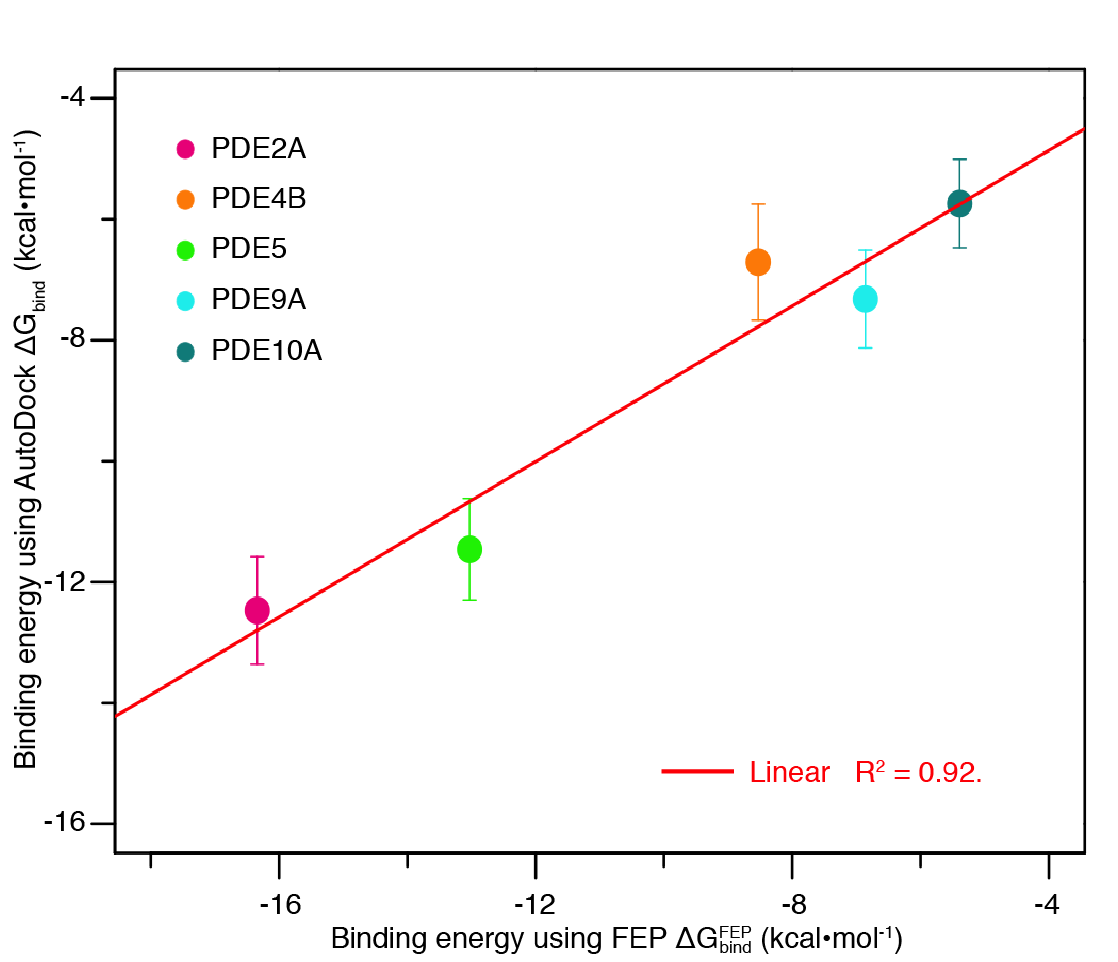 |
| --- |
| **Fig. S8.** Correlation of binding free energies calculated using FEP with those by the AutoDock energy function. There is a strong linear relationship between $\Delta G_{bind}^{FEP}$ and $\Delta G_{bind}$, indicating that the free-energy calculations by the AutoDock function are reliable. |

S12. Summary of calculated binding energies

| 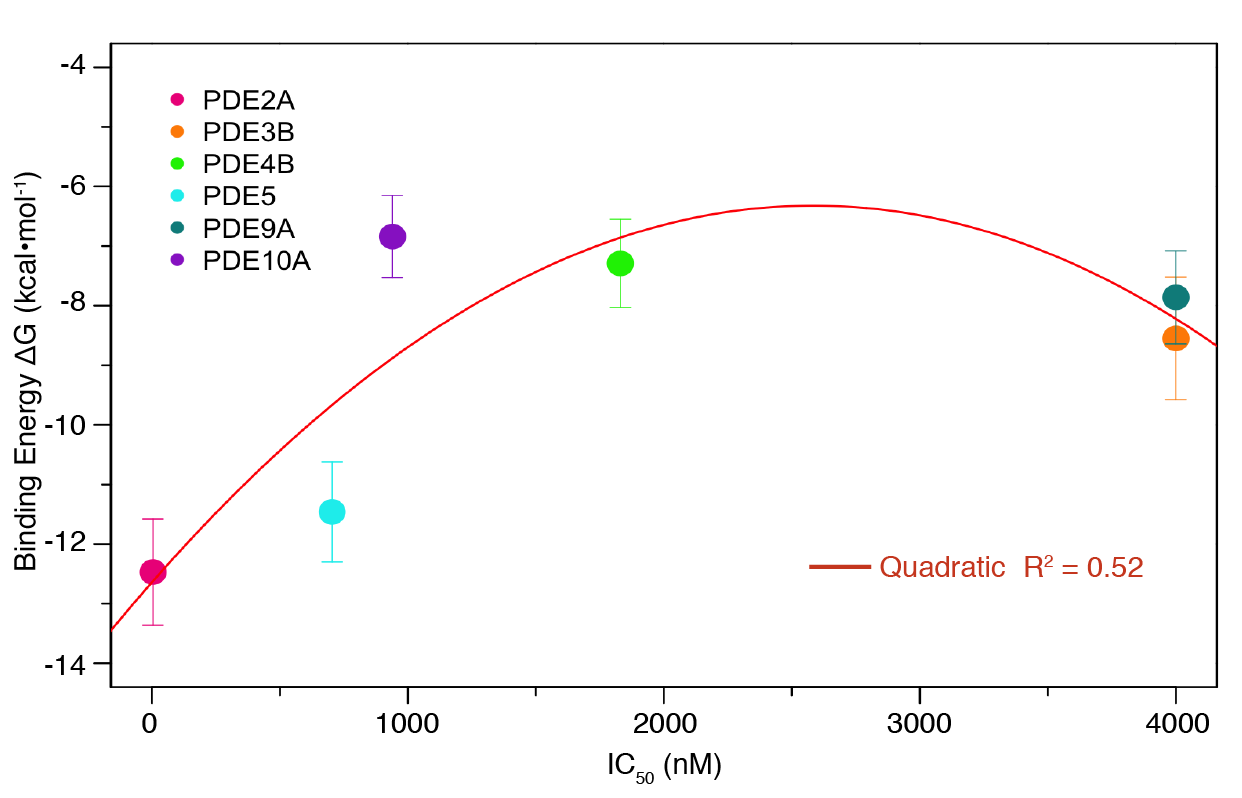 |
| --- |
| **Fig. S9.** Scatter plot of IC_50_ values versus the minimal binding energies of PDE systems. Quadratic polynomial regression is applied to these data. |

| Table S3 | | | | | | | | | | | | | | | |
| --- | --- | --- | --- | --- | --- | --- | --- | --- | --- | --- | --- | --- | --- | --- | --- |
| Summary of binding energies ΔG, standard deviations (SDs) and minimal-RMSDs of all successful MD trajectories. | | | | | | | | | | | | | | | |
| PDE ID | PDE2A | | | | PDE3B | PDE4B | | PDE5 | | PDE9A | | | PDE10A | | |
| Trajectory ID | 1-D | 2-B | 4-C | 6-D | 8-C | 2-D | 8-D | 3-A | 8-B | 2-A | 4-A | 6-D | 3-B | 4-C | 5-B |
| ΔG (kcal∙mol^-1^)^a^ | -12.47 | -8.58 | -9.78 | -5.59 | -8.55 | -7.29 | -6.71 | -8.32 | -11.46 | -7.32 | -7.86 | -7.73 | -5.74 | -6.84 | -6.20 |
| SD (kcal∙mol^-1^) | 0.89 | 0.75 | 1.05 | 0.69 | 1.03 | 0.74 | 0.96 | 0.79 | 0.84 | 0.81 | 0.78 | 0.83 | 0.73 | 0.69 | 0.95 |
| RMSD (Å) | 0.66 | 7.32 | 7.12 | 6.72 | 12.81 | 9.68 | 7.91 | 5.47 | 0.79 | 7.45 | 10.28 | 9.30 | 6.37 | 6.82 | 10.42 |
| ^a^ Exponential average binding energy of the snapshot inhibitors which RMSDs are within 2 Å of the minimal RMSDs (see also Subsection S9). | | | | | | | | | | | | | | | |

| 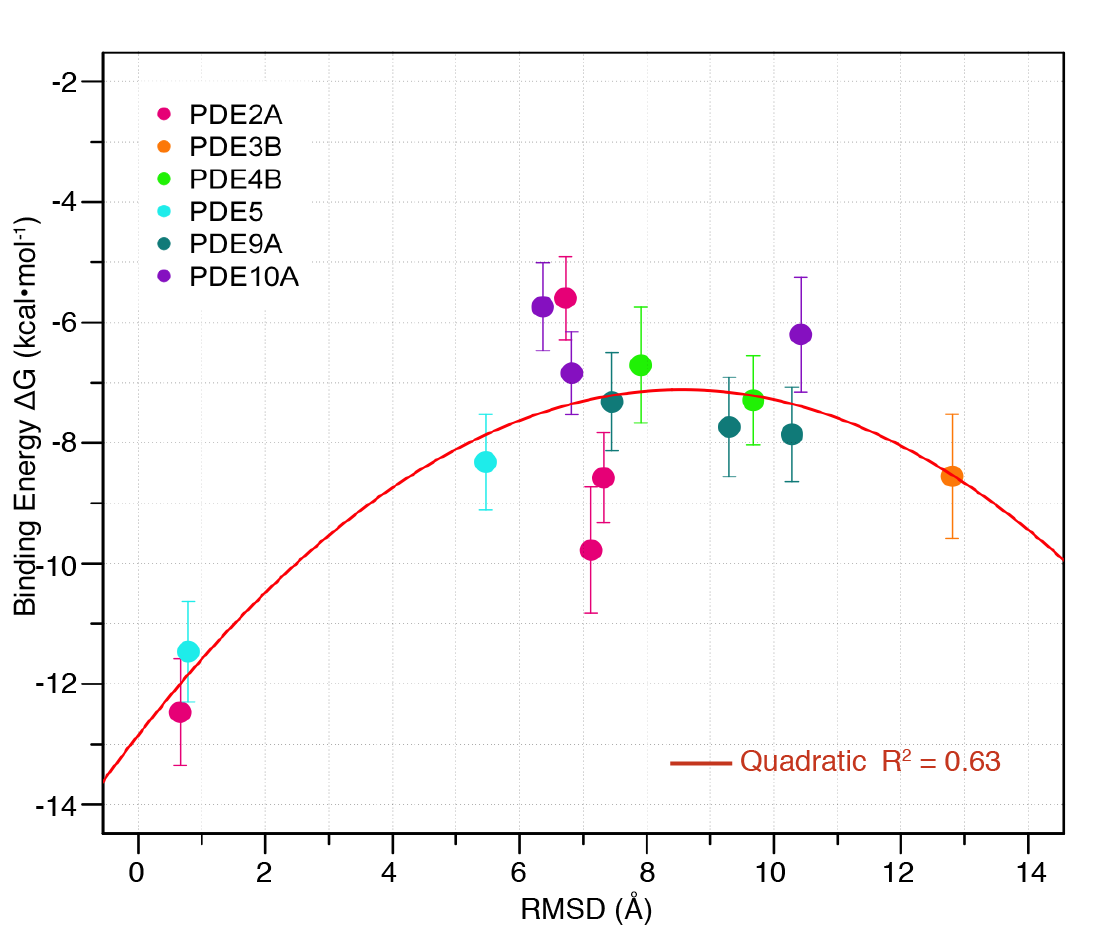 |
| --- |
| **Fig. S10.** Scatter plot of RMSDs versus binding energy of minimal-RMSD poses for all successful MD trajectories. There exists a quadratic relationship between RMSDs and binding energy values of minimal-RMSD poses for all successful MD trajectories. |

As showed in Fig. S10 and Table S3, the standard deviations of the binding energies are in the range from 0.69 to 1.05 kcal∙mol^-1^. So all the standard deviations are in the magnitude of ~1 kcal∙mol^-1^.

S13. Binding pathways of BAY60-7550 in successful MD trajectories

For each of the successful MD trajectories, we showed the binding pathway as in Fig. 6: (A) the global 3D energy landscape of the specific PDE; (B) The binding pathway in the 3D energy landscape; (C) The global energy landscape in the 2D map; (D) The binding pathway in the 2D map. The binding pathway (red lines) and time (red numbers) are shown on the energy landscape. The binding energy values are displayed as rainbow colors and elevations (only on the 3D energy landscapes). All figures are produced by the PLOT3D and FIELDS packages of R3.3.3.[[15-17](#_ENREF_15)]


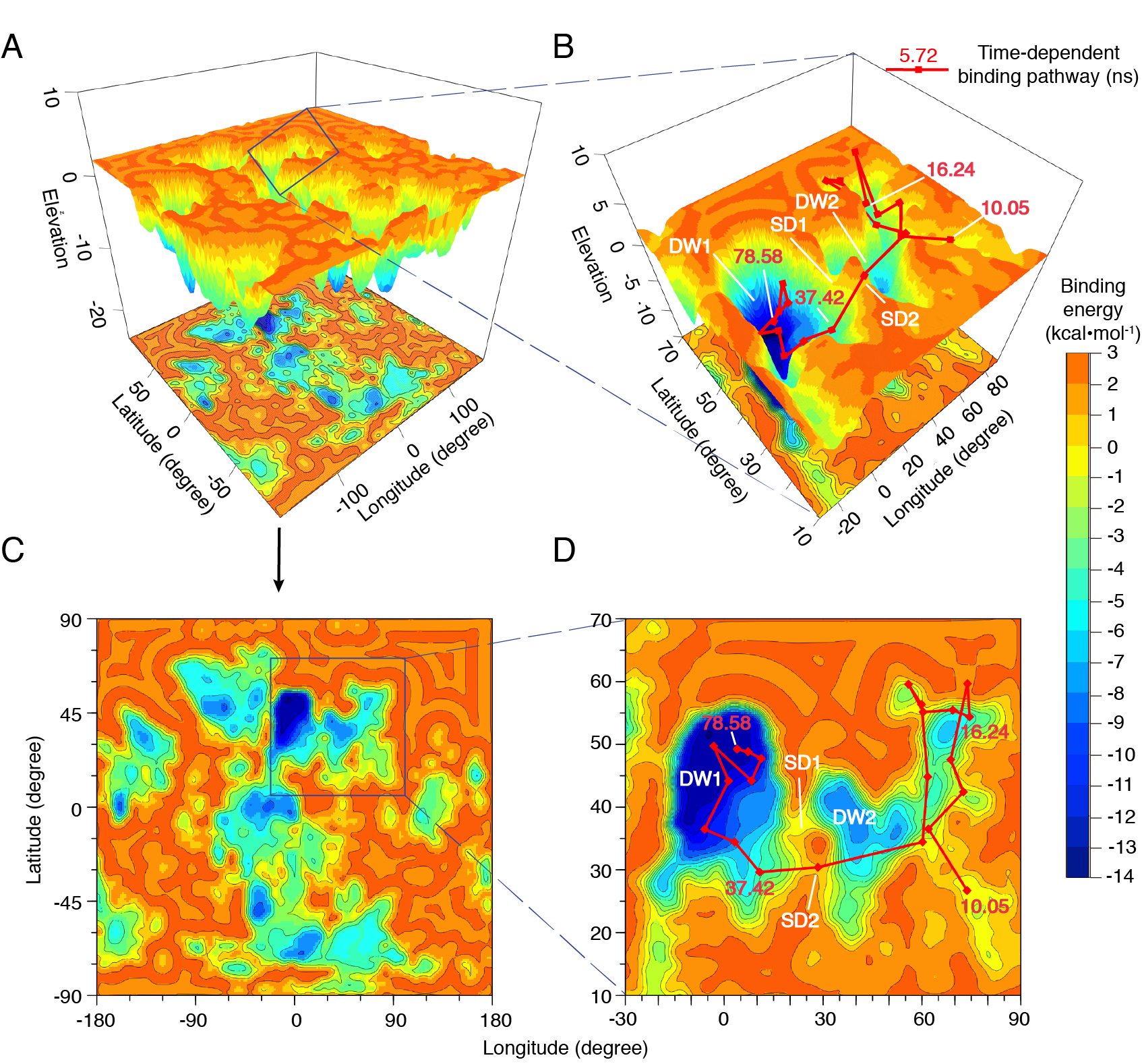


**Fig. S11.** Binding pathway of PDE2A:Traj. 2-B. This binding pathway climbs over the saddle SD2 to the energy well DW1. The energy well DW2 appears to act as a metastable area.


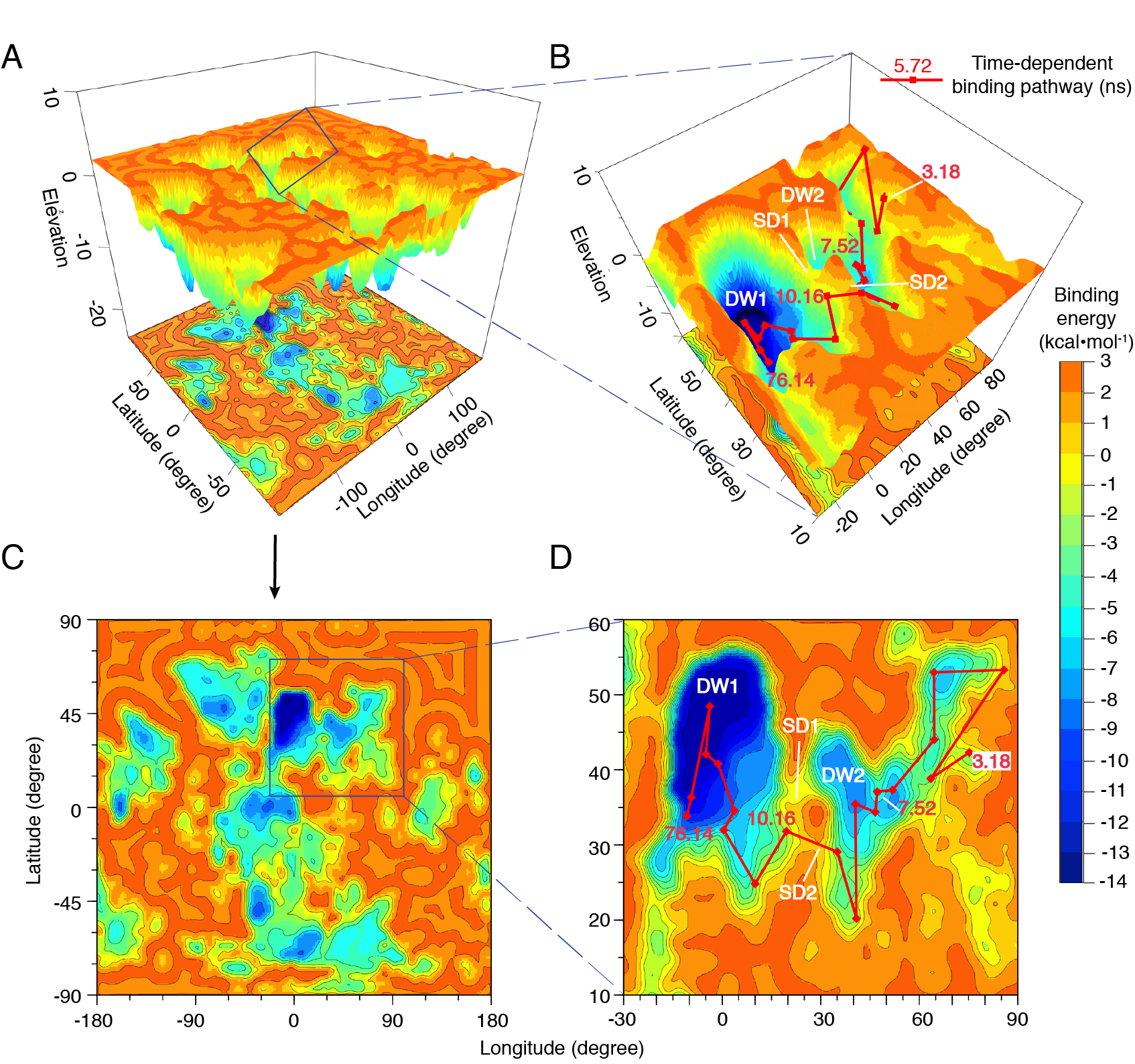


**Fig. S12.** Binding pathway of PDE2A:Traj. 4-C. This binding pathway climbs over the saddle SD2 to the energy well DW1. The energy well DW2 appears to act as a metastable area.


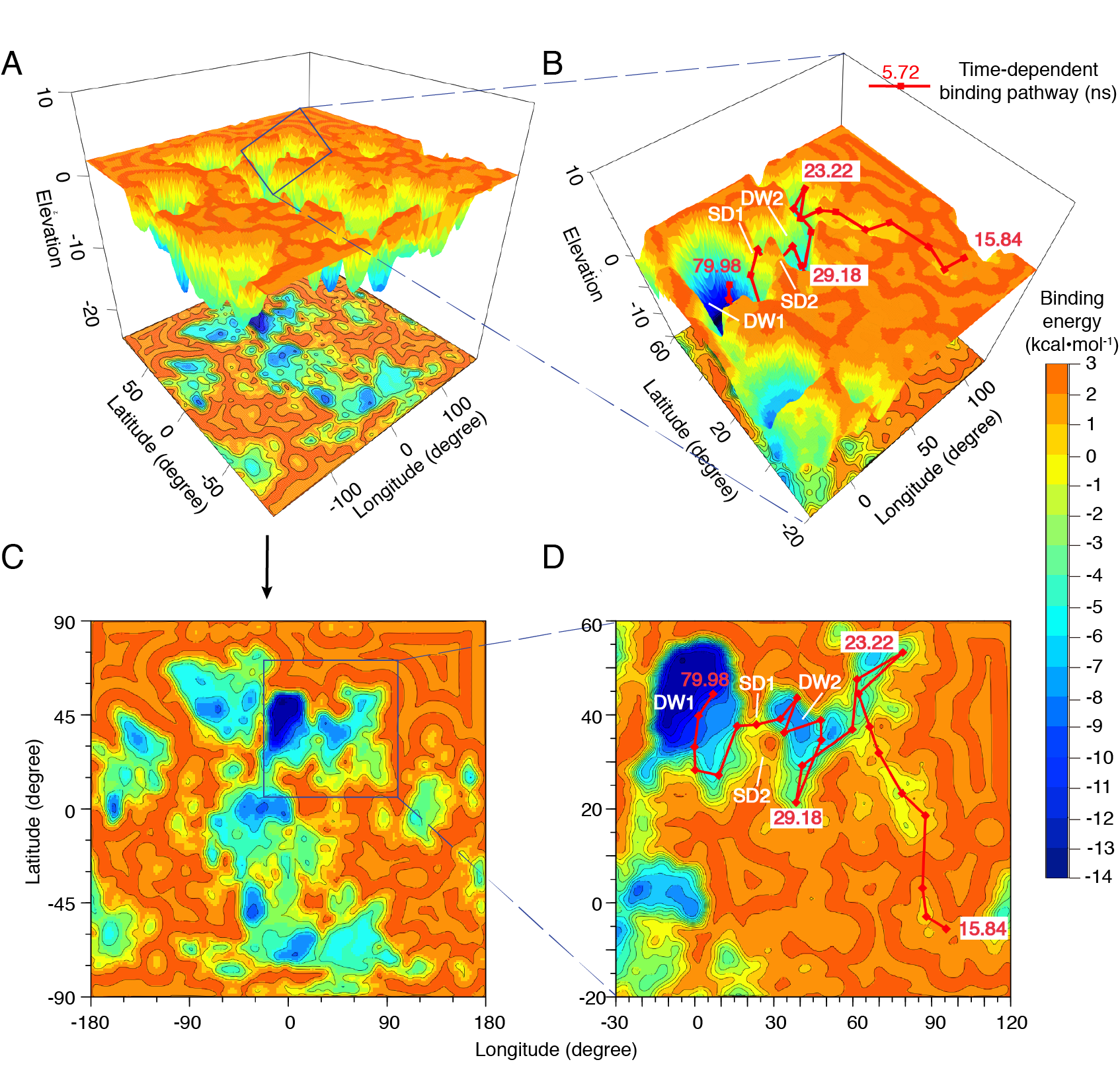


**Fig. S13.** Binding pathway of PDE2A:Traj. 6-B. This binding pathway climbs over the saddle SD1 to the energy well DW1. The energy well DW2 appears to acts as a metastable area.


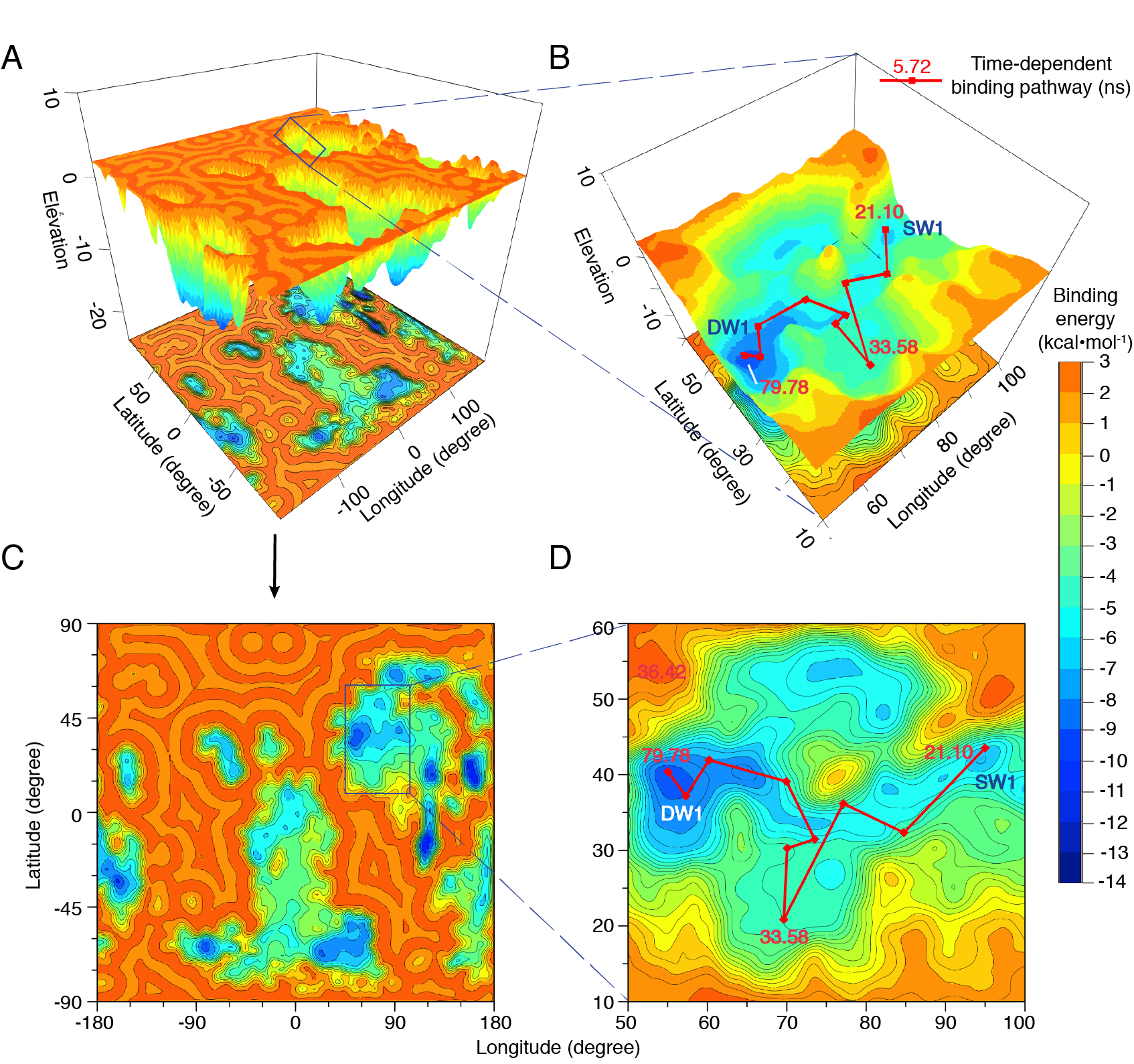


**Fig. S14.** Binding pathway of PDE3B:Traj. 8-C**.** This binding pathway is from SW1 to DW1.


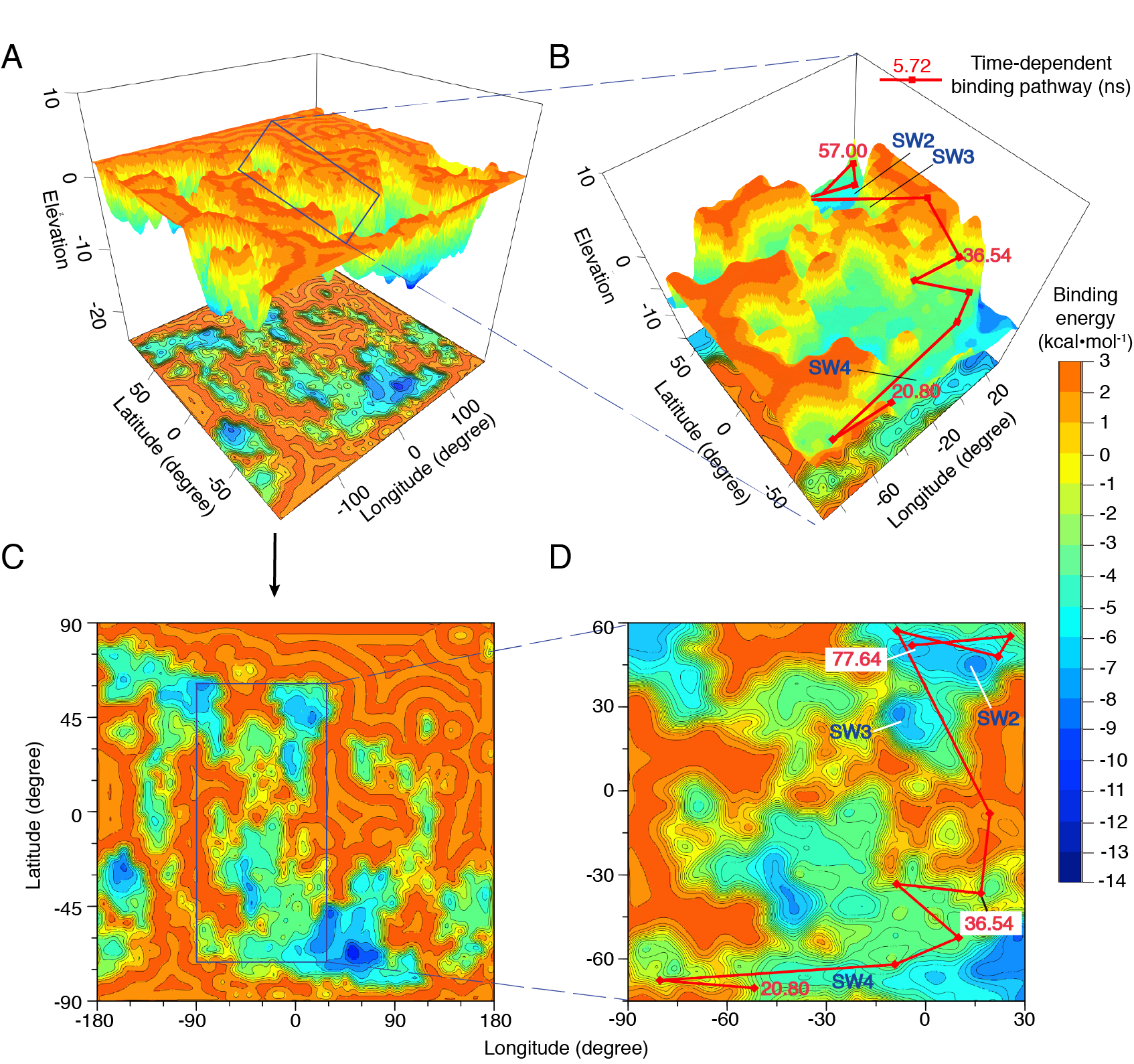


**Fig. S15.** Binding pathway of PDE4B:Traj. 2-D. This binding pathway walks through the energy well SW4 and climbs over energy barriers to reach the well SW2.


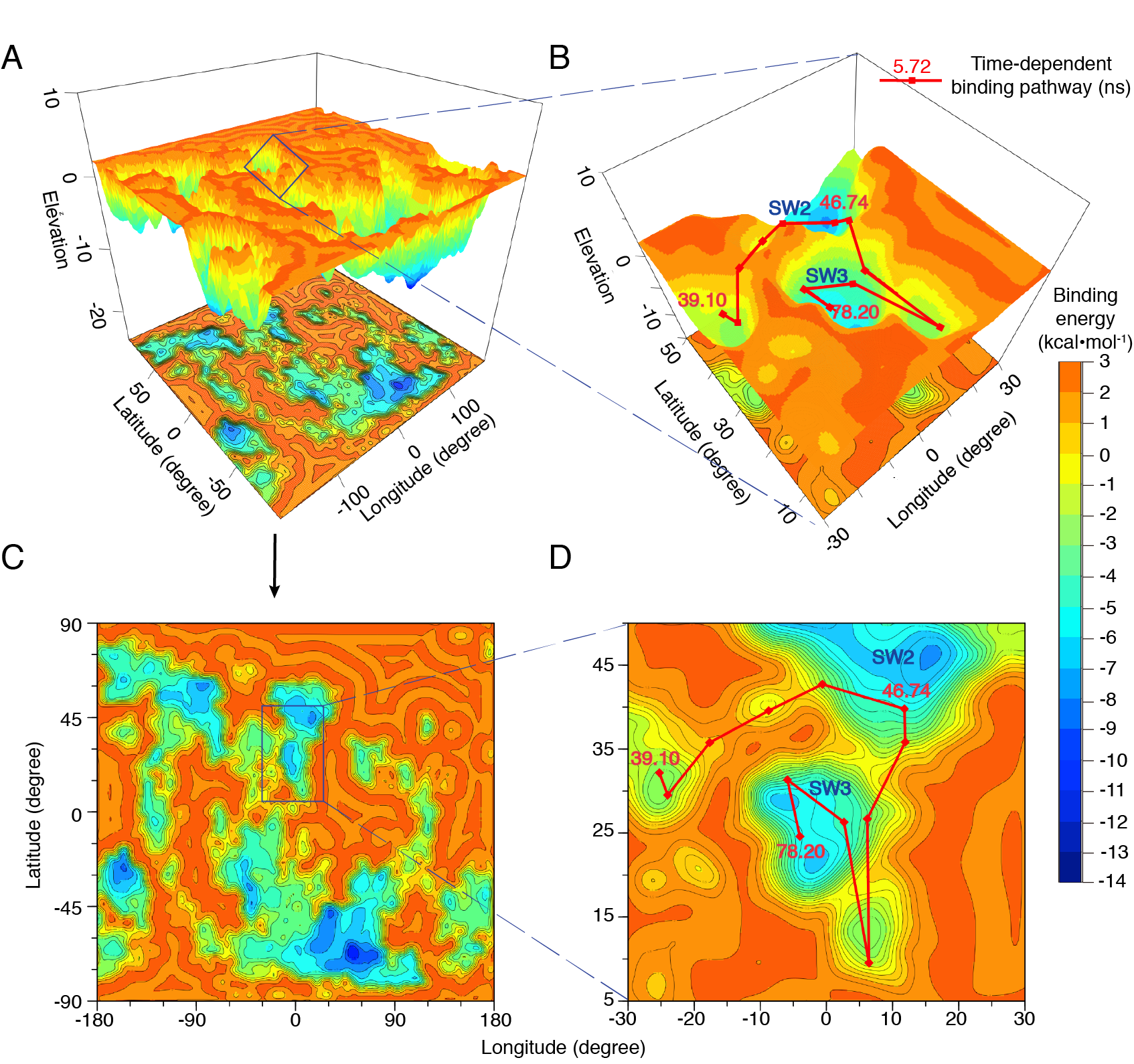


**Fig. S16.** Binding pathway of PDE4B:Traj. 8-D. This binding pathway climbs over the energy barrier between the energy wells SW2 and SW3, and then reaches the energy well SW3.


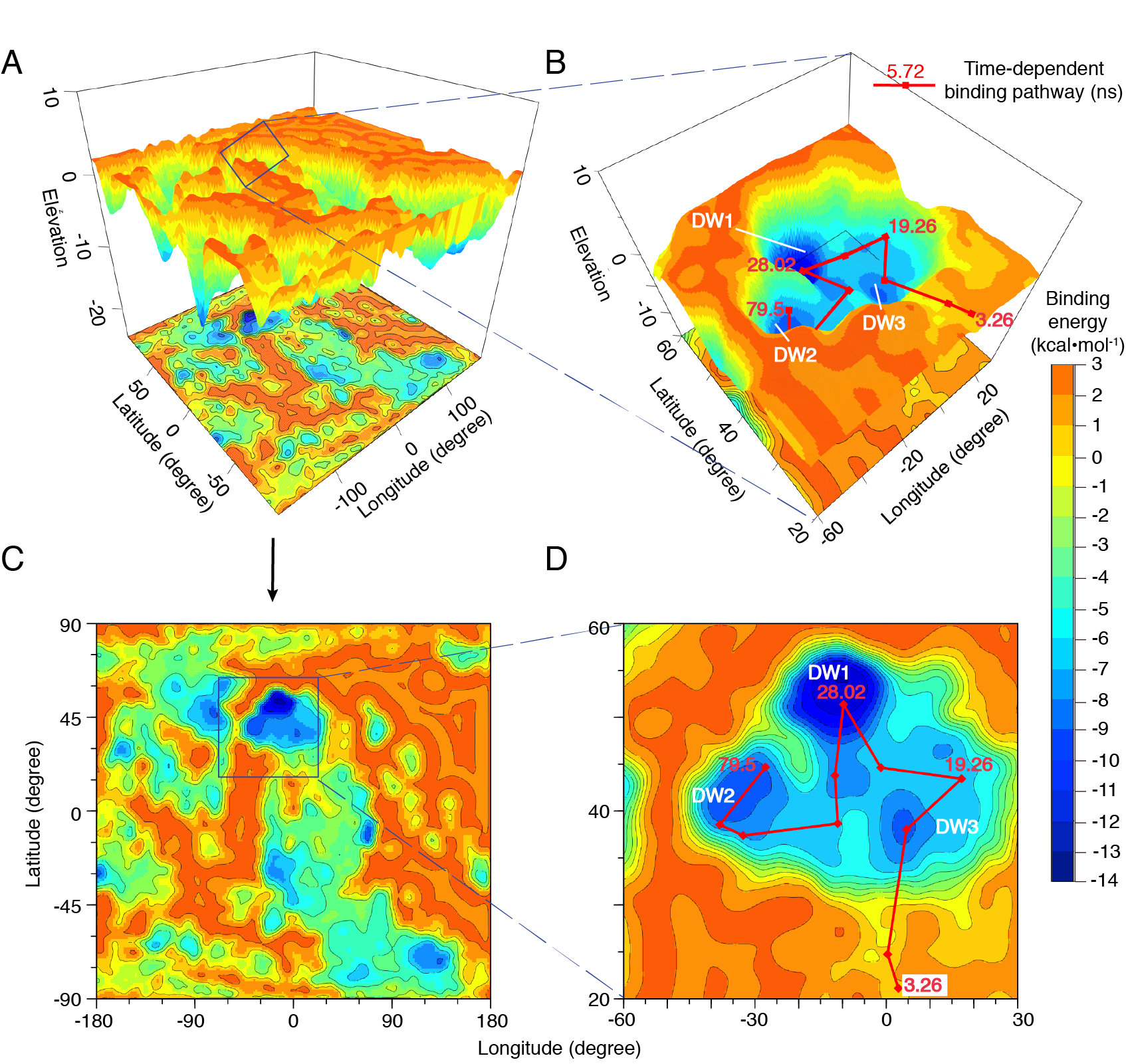


**Fig. S17.** Binding pathway of PDE5:Traj. 3-A. This binding pathway walks through the energy wells DW3 and DW1, and then reaches the energy well DW2.


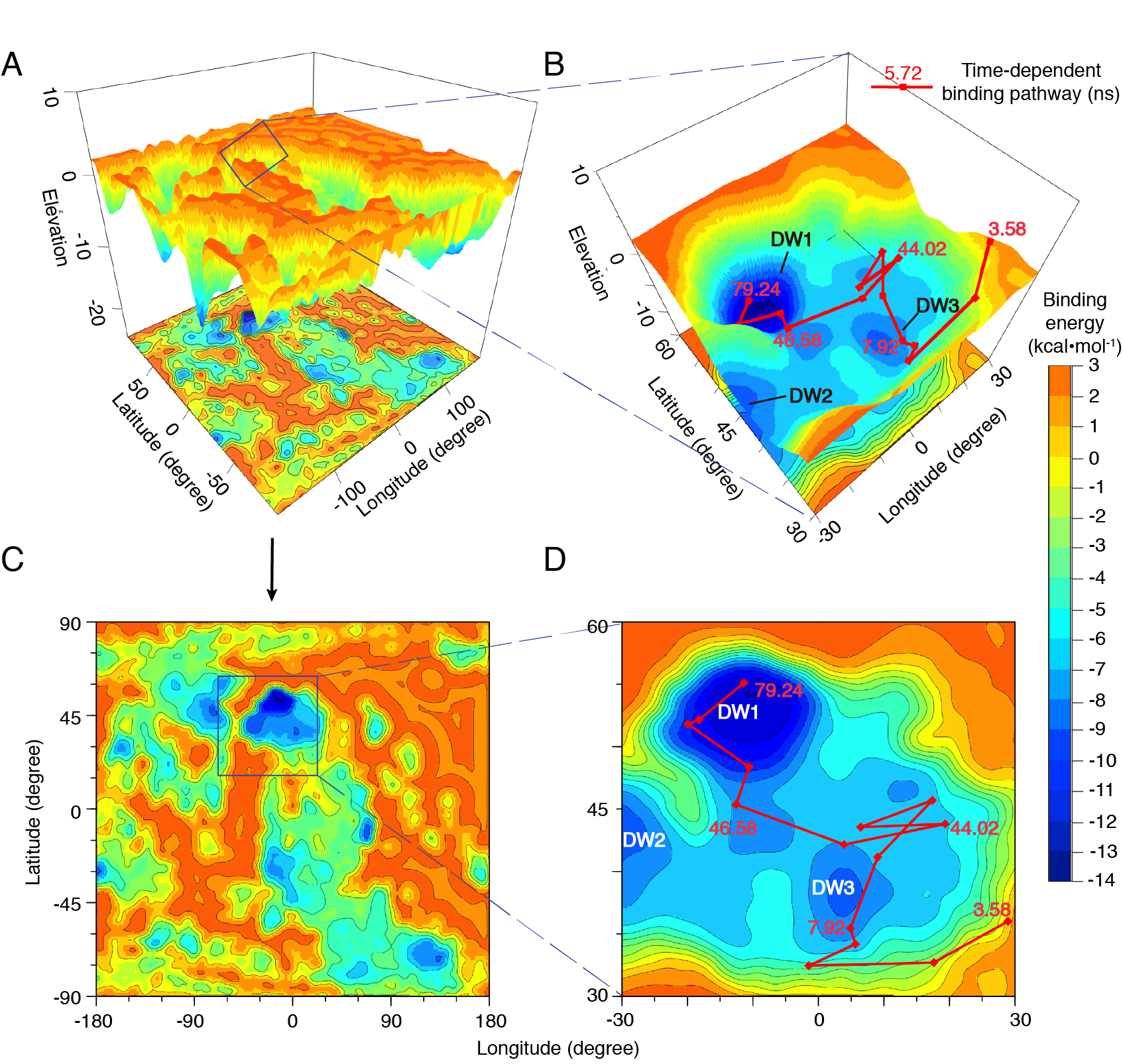


**Fig. S18.** Binding pathway of PDE5:Traj. 8-B. This binding pathway passes through the low energy barrier between the energy wells DW3 and DW1, and binds to DW1 with the native binding pose (RMSDs = 0.79 Å).


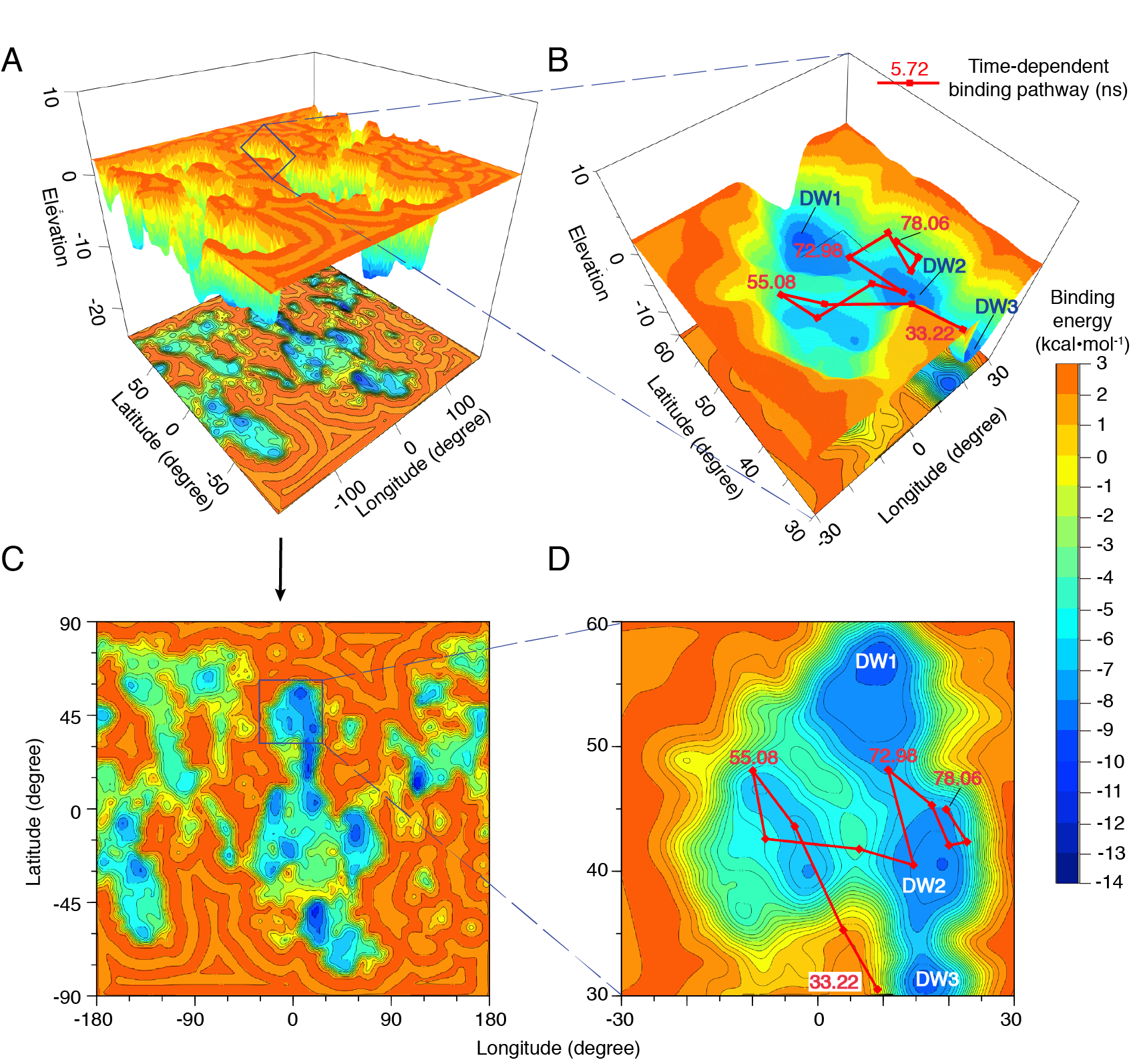


**Fig. S19.** Binding pathway of PDE9A:Traj. 2-A. This binding pathway wanders between the energy wells DW2 and DW1, due to the low-energy barrier between these two wells.


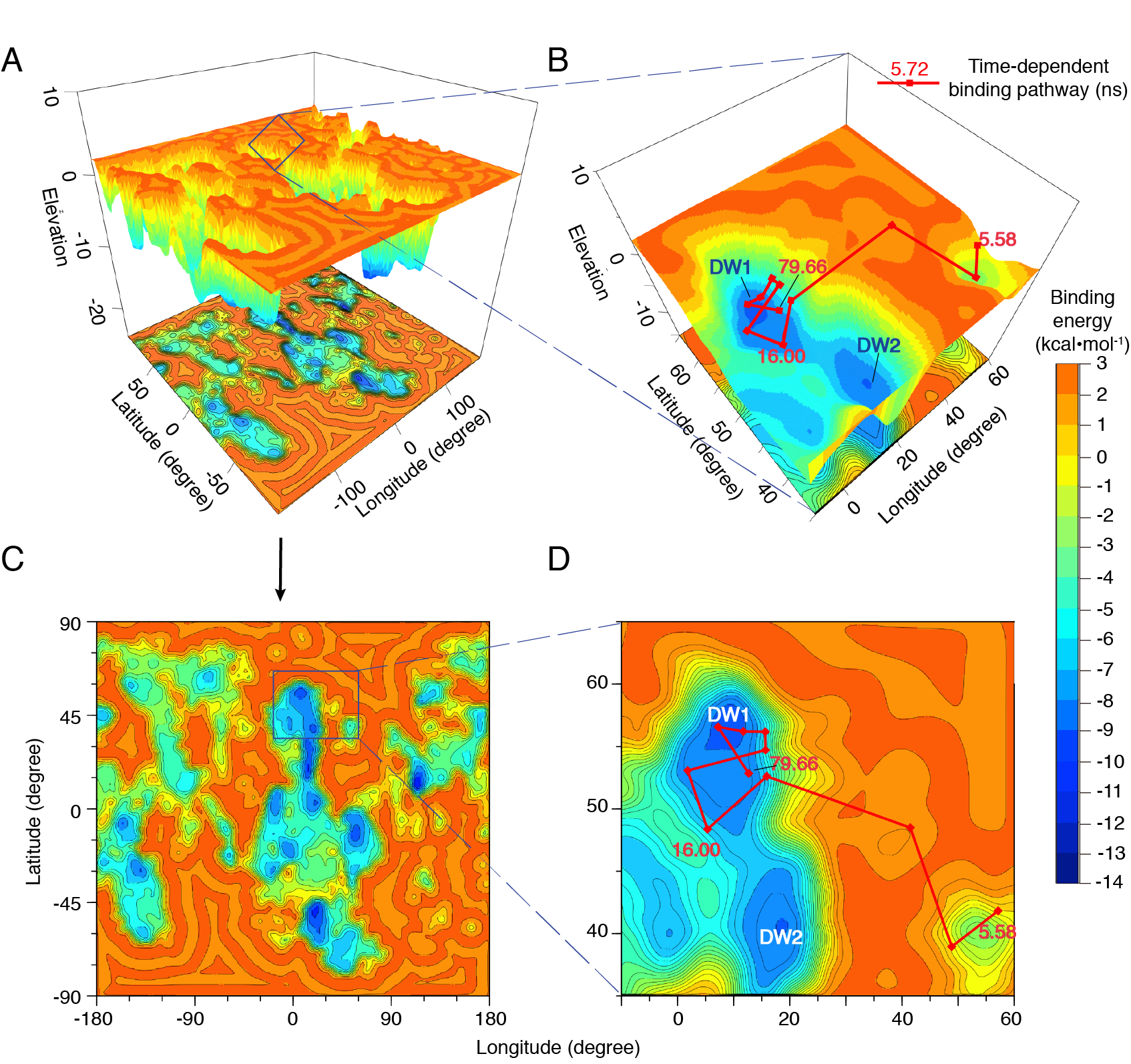


**Fig. S20.** Binding pathway of PDE9A:Traj. 4-A. This binding pathway climbs a high and large energy barrier to reach well DW1.


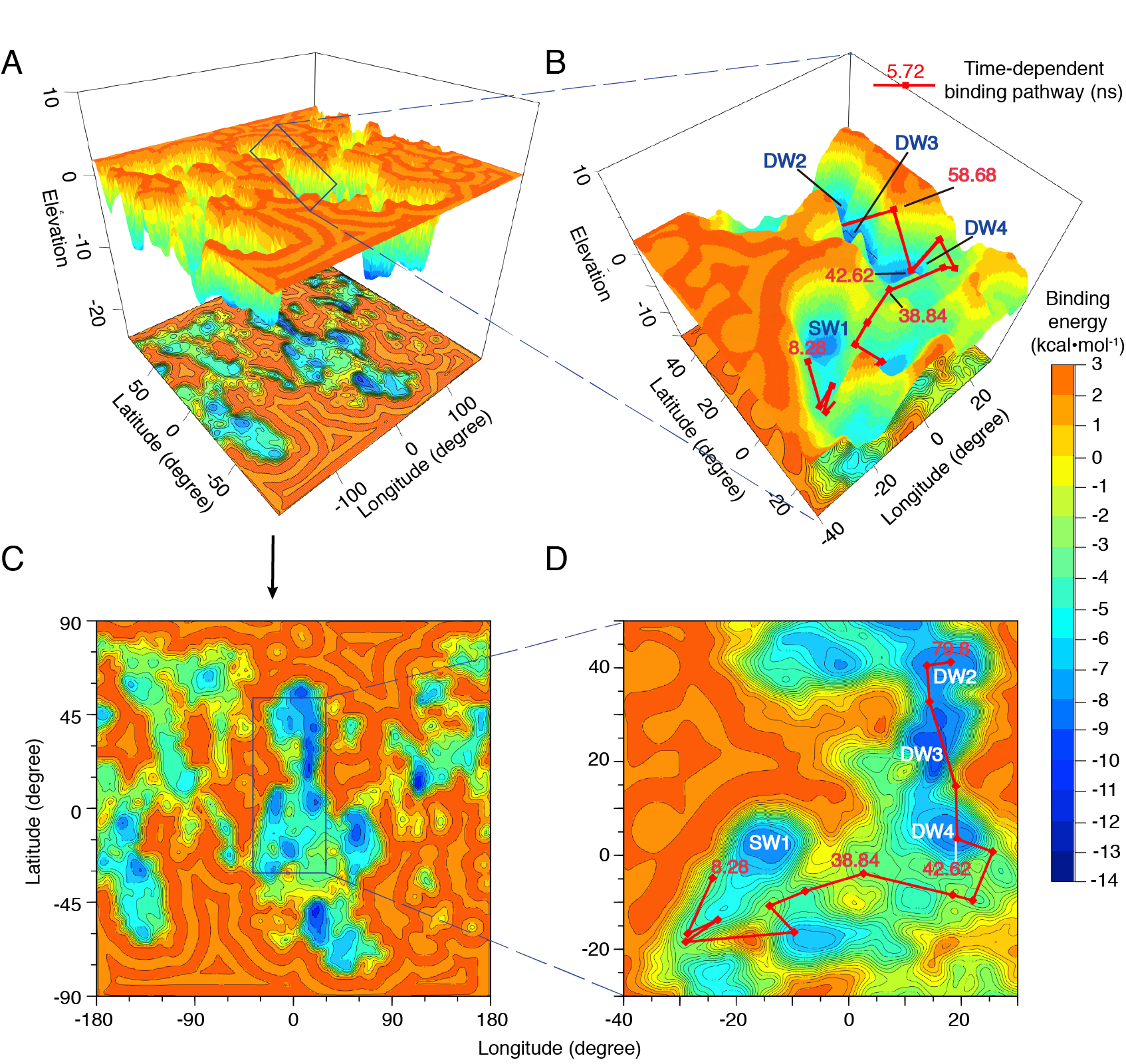


**Fig. S21.** Binding pathway of PDE9A:Traj. 6-D. Along this binding pathway, the energy wells SW1 and DW2~4 act as a metastable area to guide the inhibitors to the energy well DW2.


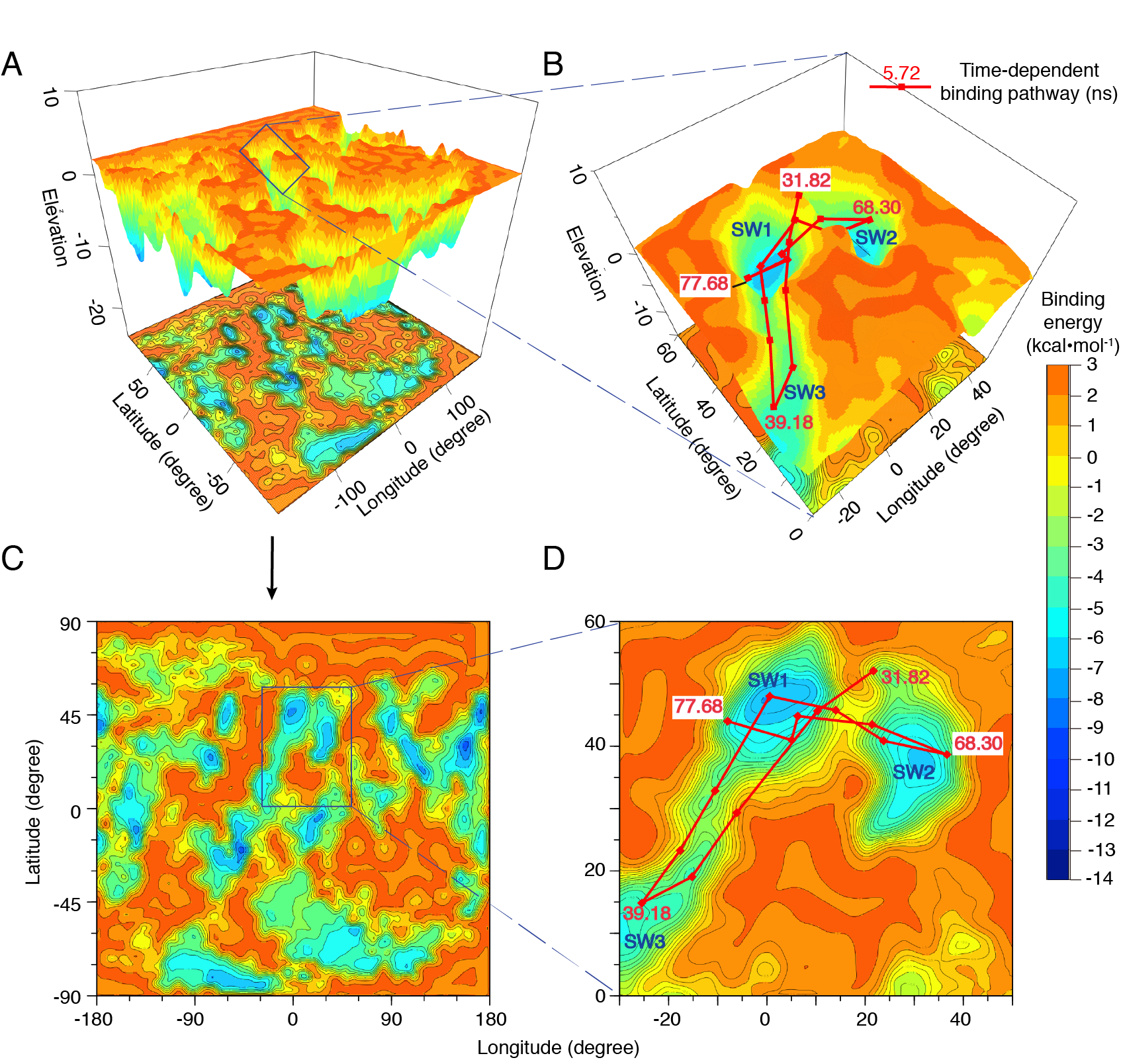


**Fig. S22.** Binding pathway of PDE10A:Traj. 3-B. This binding pathway wanders in the large regions covering the energy wells SW1~3, because the energy barriers between these wells are low.


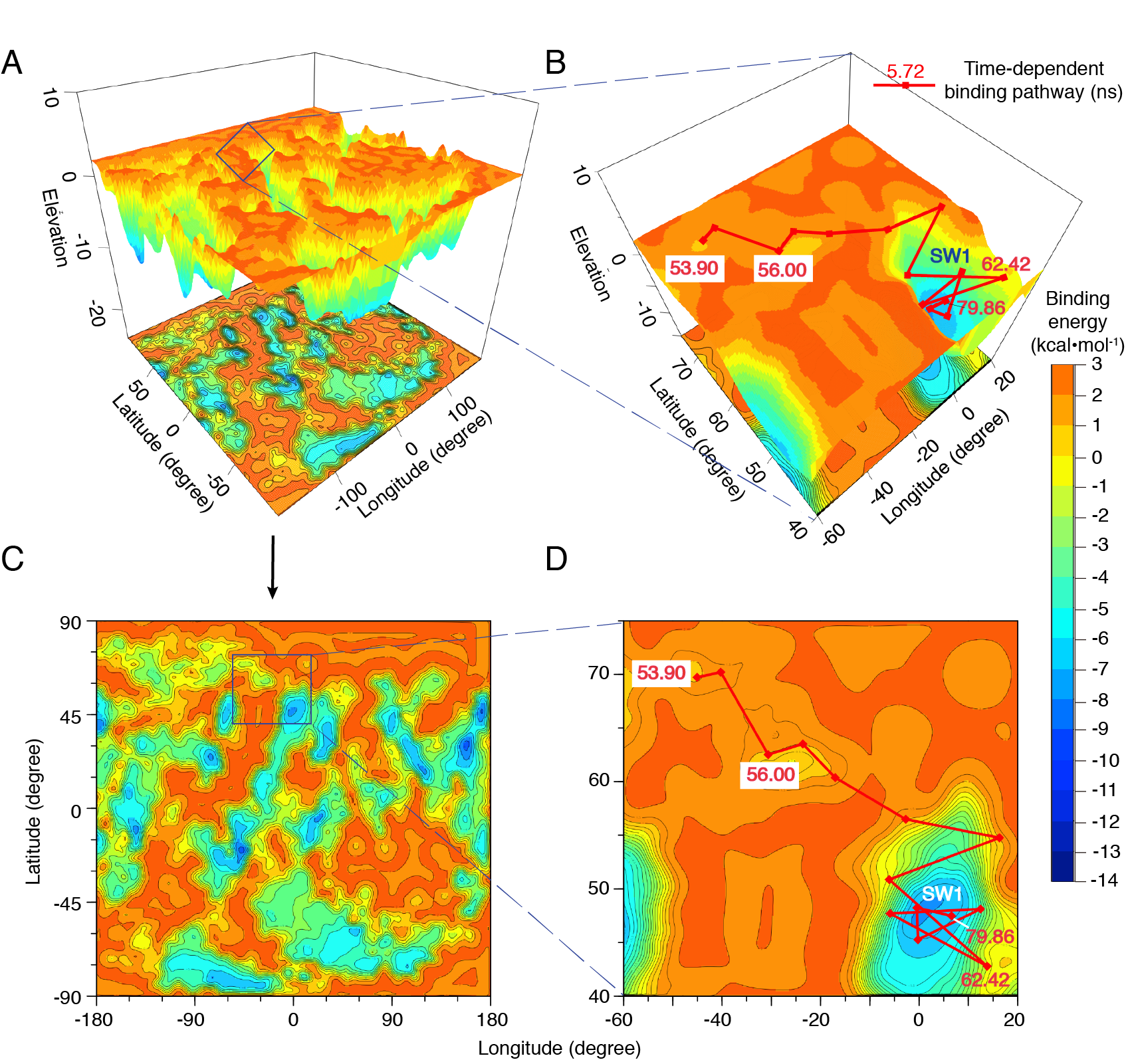


**Fig. S23.** Binding pathway of PDE10A:Traj. 4-C. This binding pathway begins very late in its MD simulation, due to the weak capability of the PDE10A catalytic pocket to associate with the inhibitor.


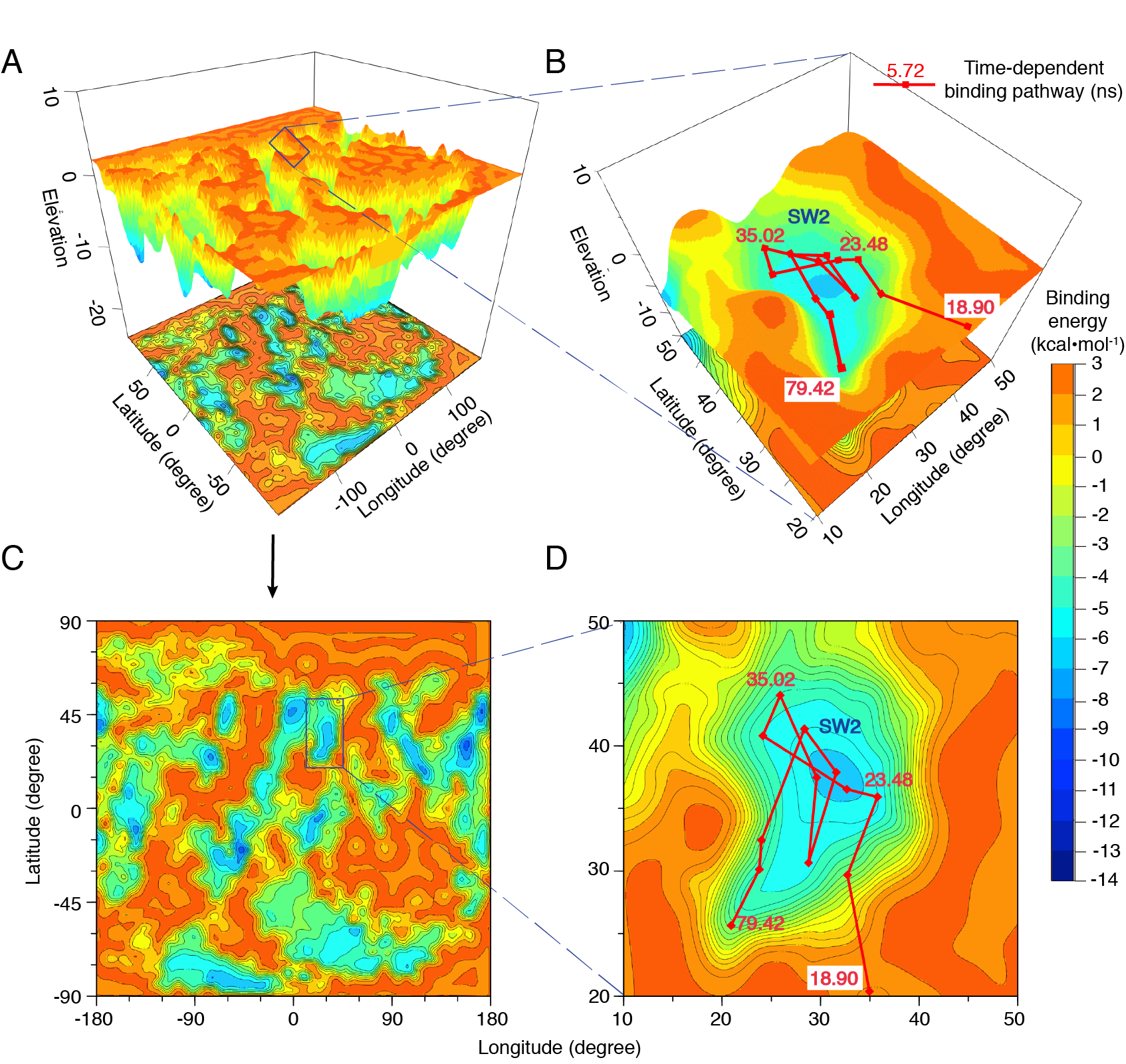


**Fig. S24.** Binding pathway of PDE10A: Traj. 5-B. This binding pathway wanders in the energy well SW2.

S14. Movie showing BAY60-7500 association with PDE2A catalytic pocket

The supplementary movie shows the spontaneous association of BAY60-7550 with the catalytic pocket in the MD trajectory PDE2A:Traj. 1-D (see also Fig. 3). In the movie, the protein PDE2A is shown as cartoon models in gray, BAY60-7550 as stick models in cyan, the H-pocket residues and F862 as stick models in magenta, and Mg^2+^ and Zn^2+^ as spheres in orange and green, respectively. For the sake of clarity, water molecules in the simulated system, hydrogen atoms of BAY60-7550 and the H-pocket residues and F862 are not shown in the movie.

References

[1] Robert X, and Gouet P, Deciphering key features in protein structures with the new ENDscript server, Nucleic Acids Res.*,* vol. 42, pp. W320-W324, 2014.

[2] Dror RO, Pan AC, Arlow DH *et al.*, Pathway and mechanism of drug binding to G-protein-coupled receptors, Proc. Natl. Acad. Sci. U. S. A.*,* vol. 108, pp. 13118-13123, 2011.

[3] Zhu J, Yang Q, Dai D *et al.*, X-ray crystal structure of phosphodiesterase 2 in complex with a highly selective, nanomolar inhibitor reveals a binding-induced pocket important for selectivity, J. Am. Chem. Soc.*,* vol. 135, pp. 11708-11711, 2013.

[4] Schrӧdinger LLC, The PyMOL molecular graphics system, 1.8 ed., 2017,https://www.pymol.org.

[5] Essmann U, Perera L, Berkowitz ML *et al.*, A smooth particle mesh Ewald method, J. Chem. Phys.*,* vol. 103, pp. 8577-8593, 1995.

[6] Martyna GJ, Tobias DJ, and Klein ML, Constant pressure molecular dynamics algorithms, J. Chem. Phys.*,* vol. 101, pp. 4177-4189, 1994.

[7] Ryckaert J-P, Ciccotti G, and Berendsen HJC, Numerical integration of the cartesian equations of motion of a system with constraints: molecular dynamics of n-alkanes, J. Comput. Phys.*,* vol. 23, pp. 327-341, 1977.

[8] Zhang Y, and Skolnick J, TM-align: a protein structure alignment algorithm based on the TM-score, Nucleic Acids Res.*,* vol. 33, pp. 2302-2309, 2005.

[9] Huey R, Morris GM, Olson AJ *et al.*, A semiempirical free energy force field with charge-based desolvation, J. Comput. Chem.*,* vol. 28, pp. 1145-52, 2007.

[10] Morris GM, Huey R, Lindstrom W *et al.*, AutoDock4 and AutoDockTools4: automated docking with selective receptor flexibility, J. Comput. Chem.*,* vol. 30, pp. 2785-2791, 2009.

[11] Weiner SJ, Kollman PA, Case DA *et al.*, A new force field for molecular mechanical simulation of nucleic acids and proteins, J. Am. Chem. Soc.*,* vol. 106, pp. 765-784, 1984.

[12] Stouten PFW, Frömmel C, Nakamura H *et al.*, An effective solvation term based on atomic occupancies for use in protein simulations, Mol. Simulat.*,* vol. 10, pp. 97-120, 1993.

[13] Wesson L, and Eisenberg D, Atomic solvation parameters applied to molecular dynamics of proteins in solution, Protein Sci.*,* vol. 1, pp. 227-35, 1992.

[14] Duchon J, Splines minimizing rotation-invariant semi-norms in sobolev spaces, in Proceedings of a Conference Held at Oberwolfach, Oberwolfach, Germany, 1976.

[15] Nychka D, Furrer R, Paige J *et al.*, FIELDs: tools for spatial data, 9 ed., Boulder, CO: University Corporation for Atmospheric Research, 2015,https://www.image.ucar.edu/~nychka/Fields.

[16] Soetaert K, PLOT3D: plotting multi-dimensional data, 1.1.1 ed., 2017,https://CRAN.R-project.org/package=plot3D.

[17] R Core Team, R: a language and environment for statistical computing, 3.3.3 ed., Vienna, Austria: R Foundation for Statistical Computing, 2017,https://www.R-project.org.

[18] Zwanzig RW, High-temperature equation of state by a perturbation method. i. nonpolar gases, J. Chem. Phys.*,* vol. 22, pp. 1420-1426, 1954.

[19] Abraham MJ, Murtola T, Schulz R *et al.*, GROMACS: High performance molecular simulations through multi-level parallelism from laptops to supercomputers, SoftwareX*,* vol. 1-2, pp. 19-25, 2015.

[20] Goga N, Rzepiela AJ, de Vries AH *et al.*, Efficient algorithms for langevin and dpd dynamics, J. Chem. Theory Comput.*,* vol. 8, pp. 3637-49, 2012.

[21] Van Gunsteren WF, and Berendsen HJC, A leap-frog algorithm for stochastic dynamics, Mol. Simulat.*,* vol. 1, pp. 173-185, 2007.

[22] Berendsen HJC, Postma JPM, van Gunsteren WF *et al.*, Molecular dynamics with coupling to an external bath, J. Chem. Phys.*,* vol. 81, pp. 3684-3690, 1984.

[23] Bennett CH, Efficient estimation of free energy differences from Monte Carlo data, J. Comput. Phys.*,* vol. 22, pp. 245-268, 1976.

[24] Parrinello M, and Rahman A, Polymorphic transitions in single crystals: a new molecular dynamics method, J. Appl. Phys.*,* vol. 52, pp. 7182-7190, 1981.

[25] Páll S, and Hess B, A flexible algorithm for calculating pair interactions on SIMD architectures, Comput. Phys. Commun.*,* vol. 184, pp. 2641-2650, 2013.

[26] Hess B, Bekker H, Berendsen HJC *et al.*, LINCS: A linear constraint solver for molecular simulations, J. Comput. Chem.*,* vol. 18, pp. 1463-1472, 1997.

[27] Beutler TC, Mark AE, van Schaik RC *et al.*, Avoiding singularities and numerical instabilities in free energy calculations based on molecular simulations, Chem. Phys. Lett.*,* vol. 222, pp. 529-539, 1994.
