# Supplementary material for: Surface Binding Energy Landscapes Affect Phosphodiesterase Isoform-Specific Inhibitor Selectivity
Source: Comput Struct Biotechnol J. 2018 Dec 28;17:101–9. doi: 10.1016/j.csbj.2018.11.009 (PMC6349013; doi:10.1016/j.csbj.2018.11.009)
Supplement: Supplementary file 3 — Topology and parameters of BAY60-7550 [file mmc3.docx]

* ----

* Parameters and topology for BAY60-7550.

* Generated on SwissParam

* ----

* ----

* Parameters.

* ----

BONDS

CR OR 363.214 1.4180

CR HCMM 342.991 1.0930

OR CB 404.019 1.3760

CB CB 401.068 1.3740

CB HCMM 381.853 1.0840

CB CR 356.737 1.4860

CR C=O 301.539 1.4920

C=O N=C 725.204 1.2900

C=O NC=O 419.491 1.3690

NC=O HNCO 479.511 1.0150

C=O C5A 393.511 1.4230

C=O O=C 931.963 1.2220

N=C NPYL 337.162 1.3370

NPYL C5A 453.459 1.3640

C5A C5B 512.256 1.3770

C5B N5B 320.682 1.3690

C5B CR 325.144 1.4690

N5B C5A 599.191 1.3130

C5A CR 322.481 1.4710

CR CR 306.432 1.5080

OR HOR 560.905 0.9720

ANGLES

CB CB CB 48.145 119.9770

CB CB OR 69.663 116.4950

CB CB HCMM 40.517 120.5710

CB CB CR 57.788 120.4190

NC=O C=O N=C 79.522 120.6970

NC=O C=O CR 70.814 112.7350

N=C C=O CR 70.382 119.7880

C=O NC=O C=O 51.024 120.2740

C=O NC=O HNCO 41.380 120.2770

NC=O C=O C5A 77.363 114.6230

NC=O C=O O=C 65.273 127.1520

C5A C=O O=C 74.556 126.4560

C=O N=C NPYL 100.464 108.5380

N=C NPYL C5A 70.598 127.7250

C5A NPYL C5A 82.904 109.5990

C=O C5A NPYL 64.769 125.3950

C=O C5A C5B 55.126 130.0650

NPYL C5A C5B 58.508 107.2550

C5A C5B N5B 74.700 111.6210

C5A C5B CR 55.845 128.0410

N5B C5B CR 68.511 120.6850

C5B N5B C5A 86.791 103.7790

NPYL C5A N5B 72.829 110.8650

NPYL C5A CR 67.288 121.8320

N5B C5A CR 62.250 127.6100

OR CR HCMM 56.205 108.5770

HCMM CR HCMM 37.134 108.8360

CB OR CR 77.363 102.8460

CB CR C=O 72.757 109.8330

CB CR HCMM 45.122 109.4910

C=O CR HCMM 46.778 108.3850

C5B CR HCMM 44.763 110.4570

C5A CR CR 72.397 110.0580

C5A CR HCMM 44.691 110.4670

CR CR CR 61.243 109.6080

CR CR HCMM 45.770 110.5490

CR CR OR 71.390 108.1330

CR OR HOR 57.069 106.5030

CB CR CR 54.406 108.6170

DIHEDRALS

CB CB CB CB 3.500 2 180.00

CB CB CB CR 3.500 2 180.00

CB CB CB HCMM 3.500 2 180.00

CB CB OR CR 2.191 2 180.00

CB OR CR HCMM 0.053 3 0.00

CB CB CB OR 3.500 2 180.00

CB CB CR C=O 0.100 3 0.00

CB CB CR HCMM -0.210 2 180.00

CB CB CR HCMM 0.196 3 0.00

CB CR C=O NC=O 0.200 2 180.00

CB CR C=O NC=O 0.150 3 0.00

CB CR C=O N=C 0.200 2 180.00

CB CR C=O N=C 0.150 3 0.00

C=O NC=O C=O C5A 3.000 2 180.00

C=O NC=O C=O O=C 0.388 1 0.00

C=O NC=O C=O O=C -0.292 2 180.00

C=O NC=O C=O O=C -0.072 3 0.00

C=O N=C NPYL C5A 3.000 2 180.00

NC=O C=O N=C NPYL 8.000 2 180.00

NC=O C=O CR HCMM -0.206 1 0.00

NC=O C=O CR HCMM 0.346 2 180.00

NC=O C=O CR HCMM 0.043 3 0.00

NC=O C=O C5A NPYL 1.250 2 180.00

NC=O C=O C5A C5B 1.250 2 180.00

C=O NC=O C=O N=C 3.000 2 180.00

C=O NC=O C=O CR 3.000 2 180.00

C=O C5A NPYL N=C 3.000 2 180.00

C=O C5A NPYL C5A 3.000 2 180.00

C=O C5A C5B N5B 0.000 1 0.00

C=O C5A C5B CR 0.000 1 0.00

N=C C=O NC=O HNCO 3.000 2 180.00

N=C C=O CR HCMM 0.200 2 180.00

N=C C=O CR HCMM 0.150 3 0.00

N=C NPYL C5A C5B 3.000 2 180.00

N=C NPYL C5A N5B 3.000 2 180.00

N=C NPYL C5A CR 3.000 2 180.00

NPYL N=C C=O CR 0.900 2 180.00

NPYL C5A C=O O=C 1.250 2 180.00

NPYL C5A C5B N5B 3.500 2 180.00

NPYL C5A C5B CR 3.500 2 180.00

NPYL C5A N5B C5B 3.500 2 180.00

NPYL C5A CR CR 0.000 1 0.00

NPYL C5A CR HCMM 0.000 1 0.00

C5A C=O NC=O HNCO 3.000 2 180.00

C5A NPYL C5A N5B 2.000 2 180.00

C5A NPYL C5A CR 2.000 2 180.00

C5A C5B N5B C5A 3.500 2 180.00

C5A C5B CR HCMM 0.000 1 0.00

C5B C5A C=O O=C 1.250 2 180.00

C5B C5A NPYL C5A 2.000 2 180.00

C5B N5B C5A CR 3.500 2 180.00

N5B C5B CR HCMM 0.000 1 0.00

N5B C5A CR CR 0.000 1 0.00

N5B C5A CR HCMM 0.000 1 0.00

C5A N5B C5B CR 3.500 2 180.00

C5A CR CR CR 0.150 3 0.00

C5A CR CR OR 0.150 3 0.00

C5A CR CR HCMM 0.150 3 0.00

CB CR CR CR 0.150 3 0.00

CB CR CR HCMM 0.195 3 0.00

CB CB CR CR 0.225 2 180.00

OR CB CB HCMM 3.500 2 180.00

OR CB CB OR 3.500 2 180.00

CR CB CB HCMM 3.500 2 180.00

CR C=O NC=O HNCO -0.147 1 0.00

CR C=O NC=O HNCO 2.902 2 180.00

CR C=O NC=O HNCO 0.671 3 0.00

O=C C=O NC=O HNCO 0.718 1 0.00

O=C C=O NC=O HNCO 2.487 2 180.00

O=C C=O NC=O HNCO -0.227 3 0.00

CR CR CR HCMM 0.320 1 0.00

CR CR CR HCMM -0.315 2 180.00

CR CR CR HCMM 0.132 3 0.00

CR CR OR HOR 0.135 2 180.00

CR CR OR HOR 0.118 3 0.00

CR CR CR CR 0.051 1 0.00

CR CR CR CR 0.341 2 180.00

CR CR CR CR 0.166 3 0.00

OR CR CR CR -0.344 1 0.00

OR CR CR CR 0.879 2 180.00

OR CR CR CR 0.238 3 0.00

OR CR CR HCMM -0.327 1 0.00

OR CR CR HCMM 0.536 2 180.00

OR CR CR HCMM 0.140 3 0.00

HCMM CB CB HCMM 3.500 2 180.00

HCMM CR CR HCMM 0.142 1 0.00

HCMM CR CR HCMM -0.693 2 180.00

HCMM CR CR HCMM 0.157 3 0.00

HCMM CR OR HOR 0.298 1 0.00

HCMM CR OR HOR -0.138 2 180.00

HCMM CR OR HOR 0.173 3 0.00

IMPROPER

CB CB CB OR 3.454 0 0.00

CB CB CB HCMM 1.079 0 0.00

CB CB CB CR 2.879 0 0.00

CR C=O CB HCMM 0.000 0 0.00

C=O N=C CR NC=O 9.356 0 0.00

NC=O C=O C=O HNCO -2.159 0 0.00

C=O C5A NC=O O=C 9.356 0 0.00

C5A NPYL C=O C5B 3.598 0 0.00

C5B N5B C5A CR 2.879 0 0.00

NPYL C5A N=C C5A 1.439 0 0.00

C5A N5B NPYL CR 3.598 0 0.00

CR CR C5A CR 0.000 0 0.00

CR CR C5A HCMM 0.000 0 0.00

CR OR CR CR 0.000 0 0.00

CR OR CR HCMM 0.000 0 0.00

CR CR CR HCMM 0.000 0 0.00

CR CB CR HCMM 0.000 0 0.00

CB CB CR CB 2.879 0 0.00

CR HCMM OR HCMM 0.000 0 0.00

CR HCMM C5B HCMM 0.000 0 0.00

CR HCMM CR HCMM 0.000 0 0.00

NONBONDED nbxmod 5 atom cdiel shift vatom vdistance vswitch -

cutnb 14.0 ctofnb 12.0 ctonnb 10.0 eps 1.0 e14fac 1.0 wmin 1.5

CB 0.000000 -0.070000 1.992400

C=O 0.000000 -0.110000 2.000000

NC=O 0.000000 -0.200000 1.850000

N=C 0.000000 -0.200000 1.850000

NPYL 0.000000 -0.090000 1.720000

C5A 0.000000 -0.050000 2.040000

C5B 0.000000 -0.050000 2.040000

N5B 0.000000 -0.200000 1.850000

CR 0.000000 -0.055000 2.175000 0.000000 -0.010000 1.900000

OR 0.000000 -0.152100 1.770000

O=C 0.000000 -0.120000 1.700000 0.000000 -0.120000 1.400000

HCMM 0.000000 -0.022000 1.320000

HNCO 0.000000 -0.046000 0.224500

HOR 0.000000 -0.046000 0.224500

* ----

* Topology.

* ----

MASS 201 CB 12.011000

MASS 202 C=O 12.011000

MASS 203 NC=O 14.006700

MASS 204 N=C 14.006700

MASS 205 NPYL 14.006700

MASS 206 C5A 12.011000

MASS 207 C5B 12.011000

MASS 208 N5B 14.006700

MASS 209 CR 12.011000

MASS 210 OR 15.999400

MASS 211 O=C 15.999400

MASS 212 HCMM 1.007940

MASS 213 HNCO 1.007940

MASS 214 HOR 1.007940

AUTOGENERATE ANGLES DIHE

DEFA FIRS NONE LAST NONE

RESI BAY -0.000

GROUP

ATOM CBC CB 0.0825

ATOM CAN CB -0.1500

ATOM CBB CB 0.0825

ATOM CAM CB -0.1500

ATOM CAL CB -0.1500

ATOM CAY CB -0.1435

ATOM CBA C=O 0.4490

ATOM NAU NC=O -0.4900

ATOM CBE C=O 0.7150

ATOM NAT N=C -0.6520

ATOM NBI NPYL 0.5052

ATOM CBF C5A -0.2366

ATOM CAZ C5B 0.0462

ATOM NAS N5B -0.5653

ATOM CBD C5A 0.0065

ATOM CAX CB -0.1435

ATOM CAJ CB -0.1500

ATOM CAH CB -0.1500

ATOM CAG CB -0.1500

ATOM CAI CB -0.1500

ATOM CAK CB -0.1500

ATOM CAB CR 0.2800

ATOM OAW OR -0.3625

ATOM OAV OR -0.3625

ATOM CAA CR 0.2800

ATOM CAR CR 0.2045

ATOM OAE O=C -0.5700

ATOM CAC CR 0.1810

ATOM CBH CR 0.1800

ATOM CBG CR 0.2800

ATOM CAD CR 0.0000

ATOM OAF OR -0.6800

ATOM CAQ CR 0.0000

ATOM CAO CR 0.0000

ATOM CAP CR 0.1435

ATOM H11 HCMM 0.0000

ATOM H21 HCMM 0.0000

ATOM H31 HCMM 0.0000

ATOM H41 HCMM 0.1500

ATOM H51 HCMM 0.0000

ATOM H61 HCMM 0.0000

ATOM H71 HCMM 0.0000

ATOM H81 HCMM 0.1500

ATOM H91 HCMM 0.1500

ATOM HA1 HCMM 0.0000

ATOM HB1 HCMM 0.0000

ATOM HC1 HNCO 0.3700

ATOM HD1 HCMM 0.0000

ATOM HE1 HCMM 0.0000

ATOM HF1 HCMM 0.0000

ATOM HG1 HCMM 0.0000

ATOM HH1 HCMM 0.0000

ATOM HI1 HCMM 0.0000

ATOM HJ1 HCMM 0.0000

ATOM HK1 HCMM 0.0000

ATOM HL1 HOR 0.4000

ATOM HM1 HCMM 0.0000

ATOM HN1 HCMM 0.0000

ATOM HO1 HCMM 0.0000

ATOM HP1 HCMM 0.0000

ATOM HQ1 HCMM 0.0000

ATOM HR1 HCMM 0.0000

ATOM HS1 HCMM 0.1500

ATOM HT1 HCMM 0.1500

ATOM HU1 HCMM 0.1500

ATOM HV1 HCMM 0.1500

ATOM HW1 HCMM 0.1500

BOND CAB OAW

BOND CAB H11

BOND CAB H21

BOND CAB H31

BOND OAW CBC

BOND CBC CAN

BOND CBC CBB

BOND CAN CAY

BOND CAN H41

BOND CBB OAV

BOND CBB CAM

BOND OAV CAA

BOND CAA H51

BOND CAA H61

BOND CAA H71

BOND CAM CAL

BOND CAM H81

BOND CAL CAY

BOND CAL H91

BOND CAY CAR

BOND CAR CBA

BOND CAR HA1

BOND CAR HB1

BOND CBA NAT

BOND CBA NAU

BOND NAU CBE

BOND NAU HC1

BOND CBE CBF

BOND CBE OAE

BOND NAT NBI

BOND NBI CBD

BOND NBI CBF

BOND CBF CAZ

BOND CAZ NAS

BOND CAZ CAC

BOND CAC HD1

BOND CAC HE1

BOND CAC HF1

BOND NAS CBD

BOND CBD CBH

BOND CBH CAQ

BOND CBH CBG

BOND CBH HG1

BOND CBG OAF

BOND CBG CAD

BOND CBG HH1

BOND CAD HI1

BOND CAD HJ1

BOND CAD HK1

BOND OAF HL1

BOND CAQ CAO

BOND CAQ HM1

BOND CAQ HN1

BOND CAO CAP

BOND CAO HO1

BOND CAO HP1

BOND CAP CAX

BOND CAP HQ1

BOND CAP HR1

BOND CAX CAJ

BOND CAX CAK

BOND CAJ CAH

BOND CAJ HS1

BOND CAH CAG

BOND CAH HT1

BOND CAG CAI

BOND CAG HU1

BOND CAI CAK

BOND CAI HV1

BOND CAK HW1

IMPH CBC CBB CAN OAW

IMPH CBB CAM CBC OAV

IMPH CAM CAL CBB H81

IMPH CAN CAY CBC H41

IMPH CAY CAL CAN CAR

IMPH CAR CBA CAY HA1

IMPH CAR CBA CAY HB1

IMPH CBA NAT CAR NAU

IMPH NAU CBE CBA HC1

IMPH CBE CBF NAU OAE

IMPH CBF NBI CBE CAZ

IMPH CAZ NAS CBF CAC

IMPH NBI CBF NAT CBD

IMPH CBD NAS NBI CBH

IMPH CBH CAQ CBD CBG

IMPH CBH CAQ CBD HG1

IMPH CBG OAF CBH CAD

IMPH CBG OAF CBH HH1

IMPH CAQ CAO CBH HM1

IMPH CAQ CAO CBH HN1

IMPH CAO CAP CAQ HO1

IMPH CAO CAP CAQ HP1

IMPH CAP CAX CAO HQ1

IMPH CAP CAX CAO HR1

IMPH CAX CAK CAP CAJ

IMPH CAJ CAH CAX HS1

IMPH CAH CAG CAJ HT1

IMPH CAG CAI CAH HU1

IMPH CAB H11 OAW H21

IMPH CAB H11 OAW H31

IMPH CAA H51 OAV H61

IMPH CAA H51 OAV H71

IMPH CAL CAY CAM H91

IMPH CAC HD1 CAZ HE1

IMPH CAC HD1 CAZ HF1

IMPH CAD HI1 CBG HJ1

IMPH CAD HI1 CBG HK1

IMPH CAI CAK CAG HV1

IMPH CAK CAI CAX HW1

IC CBC CAN CAY CAL 1.39 119.65 -1.13 120.05 1.40

IC CBC CAN CAY CAR 1.39 119.65 179.80 119.85 1.39

IC CBC CBB CAM CAL 1.39 120.25 0.08 119.49 1.40

IC CBC CBB CAM H81 1.39 120.25 -179.94 120.24 1.03

IC CBC CBB OAV CAA 1.39 116.11 179.99 111.68 1.43

IC CBC OAW CAB H11 1.36 114.02 -179.99 109.48 1.07

IC CBC OAW CAB H21 1.36 114.02 60.00 109.48 1.07

IC CBC OAW CAB H31 1.36 114.02 -59.97 109.47 1.07

IC CAN CBC CBB CAM 1.39 120.47 0.07 120.25 1.40

IC CAN CBC CBB OAV 1.39 120.47 179.77 116.11 1.37

IC CAN CBC OAW CAB 1.39 123.49 -0.07 114.02 1.43

IC CAN CAY CAL CAM 1.39 120.05 1.28 120.07 1.40

IC CAN CAY CAL H91 1.39 120.05 -178.74 119.98 1.03

IC CAN CAY CAR CBA 1.39 119.85 -75.45 113.63 1.42

IC CAN CAY CAR HA1 1.39 119.85 163.92 108.43 1.07

IC CAN CAY CAR HB1 1.39 119.85 42.48 107.03 1.07

IC CBB CBC CAN CAY 1.39 120.47 0.46 119.65 1.39

IC CBB CBC CAN H41 1.39 120.47 -179.57 120.16 1.03

IC CBB CBC OAW CAB 1.39 116.04 179.89 114.02 1.43

IC CBB CAM CAL CAY 1.40 119.49 -0.75 120.07 1.40

IC CBB CAM CAL H91 1.40 119.49 179.27 119.95 1.03

IC CBB OAV CAA H51 1.37 111.68 179.99 109.48 1.07

IC CBB OAV CAA H61 1.37 111.68 60.00 109.45 1.07

IC CBB OAV CAA H71 1.37 111.68 -60.03 109.48 1.07

IC CAM CBB CBC OAW 1.40 120.25 -179.88 116.04 1.36

IC CAM CBB OAV CAA 1.40 123.64 -0.33 111.68 1.43

IC CAM CAL CAY CAR 1.40 120.07 -179.65 120.09 1.39

IC CAL CAM CBB OAV 1.40 119.49 -179.60 123.64 1.37

IC CAL CAY CAN H41 1.40 120.05 178.90 120.20 1.03

IC CAL CAY CAR CBA 1.40 120.09 105.47 113.63 1.42

IC CAL CAY CAR HA1 1.40 120.09 -15.15 108.43 1.07

IC CAL CAY CAR HB1 1.40 120.09 -136.59 107.03 1.07

IC CAY CAN CBC OAW 1.39 119.65 -179.59 123.49 1.36

IC CAY CAL CAM H81 1.40 120.07 179.27 120.26 1.03

IC CAY CAR CBA NAU 1.39 113.63 -95.37 121.62 1.40

IC CAY CAR CBA NAT 1.39 113.63 87.21 121.34 1.34

IC CBA NAU CBE CBF 1.40 121.08 0.26 118.36 1.34

IC CBA NAU CBE OAE 1.40 121.08 -179.32 118.52 1.23

IC CBA NAT NBI CBF 1.34 119.84 0.02 126.57 1.34

IC CBA NAT NBI CBD 1.34 119.84 178.48 126.34 1.33

IC NAU CBA NAT NBI 1.40 116.99 0.74 119.84 1.28

IC NAU CBA CAR HA1 1.40 121.62 25.26 108.42 1.07

IC NAU CBA CAR HB1 1.40 121.62 146.70 107.04 1.07

IC NAU CBE CBF NBI 1.39 118.36 0.48 117.14 1.34

IC NAU CBE CBF CAZ 1.39 118.36 179.93 136.07 1.34

IC CBE NAU CBA NAT 1.39 121.08 -0.88 116.99 1.34

IC CBE NAU CBA CAR 1.39 121.08 -178.41 121.62 1.42

IC CBE CBF NBI NAT 1.34 117.14 -0.66 126.57 1.28

IC CBE CBF NBI CBD 1.34 117.14 -179.37 107.08 1.33

IC CBE CBF CAZ NAS 1.34 136.07 -179.28 110.05 1.33

IC CBE CBF CAZ CAC 1.34 136.07 -0.01 128.13 1.54

IC NAT CBA NAU HC1 1.34 116.99 179.15 119.46 0.98

IC NAT CBA CAR HA1 1.34 121.34 -152.16 108.42 1.07

IC NAT CBA CAR HB1 1.34 121.34 -30.72 107.04 1.07

IC NAT NBI CBF CAZ 1.28 126.57 179.74 106.79 1.34

IC NAT NBI CBD NAS 1.28 126.34 179.35 110.52 1.33

IC NAT NBI CBD CBH 1.28 126.34 3.16 125.03 1.55

IC NBI NAT CBA CAR 1.28 119.84 178.28 121.34 1.42

IC NBI CBF CBE OAE 1.34 117.14 -179.96 123.11 1.23

IC NBI CBF CAZ NAS 1.34 106.79 0.20 110.05 1.33

IC NBI CBF CAZ CAC 1.34 106.79 179.48 128.13 1.54

IC NBI CBD NAS CAZ 1.33 110.52 2.03 105.53 1.33

IC NBI CBD CBH CBG 1.33 125.03 55.05 112.23 1.54

IC NBI CBD CBH CAQ 1.33 125.03 -75.25 116.04 1.55

IC NBI CBD CBH HG1 1.33 125.03 171.76 103.38 1.07

IC CBF CBE NAU HC1 1.34 118.36 -179.77 119.46 0.98

IC CBF NBI CBD NAS 1.34 107.08 -1.94 110.52 1.33

IC CBF NBI CBD CBH 1.34 107.08 -178.13 125.03 1.55

IC CBF CAZ NAS CBD 1.34 110.05 -1.35 105.53 1.33

IC CBF CAZ CAC HD1 1.34 128.13 179.99 109.46 1.07

IC CBF CAZ CAC HE1 1.34 128.13 60.02 109.45 1.07

IC CBF CAZ CAC HF1 1.34 128.13 -60.02 109.49 1.07

IC CAZ CBF CBE OAE 1.34 136.07 -0.52 123.11 1.23

IC CAZ CBF NBI CBD 1.34 106.79 1.03 107.08 1.33

IC CAZ NAS CBD CBH 1.33 105.53 178.25 124.34 1.55

IC NAS CAZ CAC HD1 1.33 121.82 -0.82 109.46 1.07

IC NAS CAZ CAC HE1 1.33 121.82 -120.79 109.45 1.07

IC NAS CAZ CAC HF1 1.33 121.82 119.17 109.49 1.07

IC NAS CBD CBH CBG 1.33 124.34 -120.63 112.23 1.54

IC NAS CBD CBH CAQ 1.33 124.34 109.07 116.04 1.55

IC NAS CBD CBH HG1 1.33 124.34 -3.92 103.38 1.07

IC CBD NAS CAZ CAC 1.33 105.53 179.32 121.82 1.54

IC CBD CBH CBG CAD 1.55 112.23 57.33 109.84 1.53

IC CBD CBH CBG OAF 1.55 112.23 177.49 108.38 1.43

IC CBD CBH CBG HH1 1.55 112.23 -62.14 110.14 1.07

IC CBD CBH CAQ CAO 1.55 116.04 -67.09 111.91 1.55

IC CBD CBH CAQ HM1 1.55 116.04 53.26 108.85 1.07

IC CBD CBH CAQ HN1 1.55 116.04 174.09 108.03 1.07

IC CAX CAJ CAH CAG 1.39 121.50 0.01 119.39 1.38

IC CAX CAJ CAH HT1 1.39 121.50 -179.96 120.33 1.03

IC CAX CAK CAI CAG 1.38 119.99 -0.59 120.92 1.39

IC CAX CAK CAI HV1 1.38 119.99 179.41 119.54 1.03

IC CAX CAP CAO CAQ 1.40 111.74 175.17 108.44 1.55

IC CAX CAP CAO HO1 1.40 111.74 55.31 109.71 1.07

IC CAX CAP CAO HP1 1.40 111.74 -64.36 110.07 1.07

IC CAJ CAX CAK CAI 1.39 118.84 0.41 119.99 1.39

IC CAJ CAX CAK HW1 1.39 118.84 -179.59 120.01 1.03

IC CAJ CAX CAP CAO 1.39 118.98 -104.05 111.74 1.54

IC CAJ CAX CAP HQ1 1.39 118.98 135.61 108.91 1.07

IC CAJ CAX CAP HR1 1.39 118.98 14.86 108.12 1.07

IC CAJ CAH CAG CAI 1.39 119.39 -0.18 119.36 1.39

IC CAJ CAH CAG HU1 1.39 119.39 179.84 120.29 1.03

IC CAH CAJ CAX CAK 1.39 121.50 -0.13 118.84 1.38

IC CAH CAJ CAX CAP 1.39 121.50 -179.96 118.98 1.40

IC CAH CAG CAI CAK 1.38 119.36 0.47 120.92 1.39

IC CAH CAG CAI HV1 1.38 119.36 -179.53 119.54 1.03

IC CAG CAH CAJ HS1 1.38 119.39 179.99 119.22 1.03

IC CAG CAI CAK HW1 1.39 120.92 179.41 120.00 1.03

IC CAI CAG CAH HT1 1.39 119.36 179.79 120.29 1.03

IC CAI CAK CAX CAP 1.39 119.99 -179.76 122.17 1.40

IC CAK CAX CAJ HS1 1.38 118.84 179.90 119.28 1.03

IC CAK CAX CAP CAO 1.38 122.17 76.13 111.74 1.54

IC CAK CAX CAP HQ1 1.38 122.17 -44.22 108.91 1.07

IC CAK CAX CAP HR1 1.38 122.17 -164.97 108.12 1.07

IC CAK CAI CAG HU1 1.39 120.92 -179.55 120.35 1.03

IC OAW CBC CAN H41 1.36 123.49 0.38 120.16 1.03

IC OAW CBC CBB OAV 1.36 116.04 -0.19 116.11 1.37

IC OAV CBB CAM H81 1.37 123.64 0.38 120.24 1.03

IC CAR CAY CAN H41 1.39 119.85 -0.18 120.20 1.03

IC CAR CAY CAL H91 1.39 120.09 0.34 119.98 1.03

IC CAR CBA NAU HC1 1.42 121.62 1.62 119.46 0.98

IC OAE CBE NAU HC1 1.23 118.52 0.65 119.46 0.98

IC CBH CBG CAD HI1 1.54 109.84 59.58 109.45 1.07

IC CBH CBG CAD HJ1 1.54 109.84 -60.44 109.46 1.07

IC CBH CBG CAD HK1 1.54 109.84 179.53 109.48 1.07

IC CBH CBG OAF HL1 1.54 108.38 -59.54 109.44 0.97

IC CBH CAQ CAO CAP 1.55 111.91 -176.61 108.44 1.54

IC CBH CAQ CAO HO1 1.55 111.91 -56.77 109.75 1.07

IC CBH CAQ CAO HP1 1.55 111.91 62.93 110.07 1.07

IC CBG CBH CAQ CAO 1.54 111.83 162.42 111.91 1.55

IC CBG CBH CAQ HM1 1.54 111.83 -77.23 108.85 1.07

IC CBG CBH CAQ HN1 1.54 111.83 43.60 108.03 1.07

IC CAD CBG CBH CAQ 1.53 109.84 -170.25 111.83 1.55

IC CAD CBG CBH HG1 1.53 109.84 -56.27 108.49 1.07

IC CAD CBG OAF HL1 1.53 109.96 60.54 109.44 0.97

IC OAF CBG CBH CAQ 1.43 108.38 -50.10 111.83 1.55

IC OAF CBG CBH HG1 1.43 108.38 63.89 108.49 1.07

IC OAF CBG CAD HI1 1.43 109.96 -59.61 109.45 1.07

IC OAF CBG CAD HJ1 1.43 109.96 -179.63 109.46 1.07

IC OAF CBG CAD HK1 1.43 109.96 60.34 109.48 1.07

IC CAQ CBH CBG HH1 1.55 111.83 70.28 110.14 1.07

IC CAQ CAO CAP HQ1 1.55 108.44 -64.47 108.90 1.07

IC CAQ CAO CAP HR1 1.55 108.44 56.28 108.15 1.07

IC CAO CAQ CBH HG1 1.55 111.91 45.62 103.88 1.07

IC CAP CAX CAJ HS1 1.40 118.98 0.06 119.28 1.03

IC CAP CAX CAK HW1 1.40 122.17 0.24 120.01 1.03

IC CAP CAO CAQ HM1 1.54 108.44 63.04 108.85 1.07

IC CAP CAO CAQ HN1 1.54 108.44 -57.79 108.04 1.07

IC H81 CAM CAL H91 1.03 120.26 -0.71 119.95 1.03

IC HG1 CBH CBG HH1 1.07 108.49 -175.74 110.14 1.07

IC HG1 CBH CAQ HM1 1.07 103.88 165.97 108.85 1.07

IC HG1 CBH CAQ HN1 1.07 103.88 -73.20 108.03 1.07

IC HH1 CBG CAD HI1 1.07 108.52 -179.96 109.45 1.07

IC HH1 CBG CAD HJ1 1.07 108.52 60.02 109.46 1.07

IC HH1 CBG CAD HK1 1.07 108.52 -60.01 109.48 1.07

IC HH1 CBG OAF HL1 1.07 109.99 179.99 109.44 0.97

IC HM1 CAQ CAO HO1 1.07 108.85 -177.12 109.75 1.07

IC HM1 CAQ CAO HP1 1.07 108.85 -57.42 110.07 1.07

IC HN1 CAQ CAO HO1 1.07 108.04 62.04 109.75 1.07

IC HN1 CAQ CAO HP1 1.07 108.04 -178.26 110.07 1.07

IC HO1 CAO CAP HQ1 1.07 109.71 175.66 108.90 1.07

IC HO1 CAO CAP HR1 1.07 109.71 -63.59 108.15 1.07

IC HP1 CAO CAP HQ1 1.07 110.07 56.00 108.90 1.07

IC HP1 CAO CAP HR1 1.07 110.07 176.75 108.15 1.07

IC HS1 CAJ CAH HT1 1.03 119.22 0.01 120.33 1.03

IC HT1 CAH CAG HU1 1.03 120.29 -0.19 120.29 1.03

IC HU1 CAG CAI HV1 1.03 120.35 0.46 119.54 1.03

IC HV1 CAI CAK HW1 1.03 119.54 -0.59 120.00 1.03

IC CBB CAN *CBC OAW 0.00 0.00 180.00 0.00 0.00

IC CAM CBC *CBB OAV 0.00 0.00 180.00 0.00 0.00

IC CAL CBB *CAM H81 0.00 0.00 180.00 0.00 0.00

IC CAY CBC *CAN H41 0.00 0.00 180.00 0.00 0.00

IC CAL CAN *CAY CAR 0.00 0.00 180.00 0.00 0.00

IC CBA CAY *CAR HA1 0.00 0.00 120.00 0.00 0.00

IC CBA CAY *CAR HB1 0.00 0.00 -120.00 0.00 0.00

IC NAT CAR *CBA NAU 0.00 0.00 180.00 0.00 0.00

IC CBE CBA *NAU HC1 0.00 0.00 180.00 0.00 0.00

IC CBF NAU *CBE OAE 0.00 0.00 180.00 0.00 0.00

IC NBI CBE *CBF CAZ 0.00 0.00 180.00 0.00 0.00

IC NAS CBF *CAZ CAC 0.00 0.00 180.00 0.00 0.00

IC CBF NAT *NBI CBD 0.00 0.00 180.00 0.00 0.00

IC NAS NBI *CBD CBH 0.00 0.00 180.00 0.00 0.00

IC CAQ CBD *CBH CBG 0.00 0.00 120.00 0.00 0.00

IC CAQ CBD *CBH HG1 0.00 0.00 -120.00 0.00 0.00

IC OAF CBH *CBG CAD 0.00 0.00 120.00 0.00 0.00

IC OAF CBH *CBG HH1 0.00 0.00 -120.00 0.00 0.00

IC CAO CBH *CAQ HM1 0.00 0.00 120.00 0.00 0.00

IC CAO CBH *CAQ HN1 0.00 0.00 -120.00 0.00 0.00

IC CAP CAQ *CAO HO1 0.00 0.00 120.00 0.00 0.00

IC CAP CAQ *CAO HP1 0.00 0.00 -120.00 0.00 0.00

IC CAX CAO *CAP HQ1 0.00 0.00 120.00 0.00 0.00

IC CAX CAO *CAP HR1 0.00 0.00 -120.00 0.00 0.00

IC CAK CAP *CAX CAJ 0.00 0.00 180.00 0.00 0.00

IC CAH CAX *CAJ HS1 0.00 0.00 180.00 0.00 0.00

IC CAG CAJ *CAH HT1 0.00 0.00 180.00 0.00 0.00

IC CAI CAH *CAG HU1 0.00 0.00 180.00 0.00 0.00

IC H11 OAW *CAB H21 0.00 0.00 120.00 0.00 0.00

IC H11 OAW *CAB H31 0.00 0.00 -120.00 0.00 0.00

IC H51 OAV *CAA H61 0.00 0.00 120.00 0.00 0.00

IC H51 OAV *CAA H71 0.00 0.00 -120.00 0.00 0.00

IC CAY CAM *CAL H91 0.00 0.00 180.00 0.00 0.00

IC HD1 CAZ *CAC HE1 0.00 0.00 120.00 0.00 0.00

IC HD1 CAZ *CAC HF1 0.00 0.00 -120.00 0.00 0.00

IC HI1 CBG *CAD HJ1 0.00 0.00 120.00 0.00 0.00

IC HI1 CBG *CAD HK1 0.00 0.00 -120.00 0.00 0.00

IC CAK CAG *CAI HV1 0.00 0.00 180.00 0.00 0.00

IC CAI CAX *CAK HW1 0.00 0.00 180.00 0.00 0.00

END
